# Leptin Neutralization Attenuates Inflammaging in Endothelial and Immune Cell Models

**DOI:** 10.1002/mco2.71082

**Published:** 2026-10-23

**Authors:** Valeria Pellegrini, Rosalba La Grotta, Alessia Metallo, Francesca Carreras, Chiara Frigé, Giulia Matacchione, Angelica Giuliani, Jacopo Sabbatinelli, Fabiola Olivieri, Francesca Rispoli, Lorenzo Drago, Cesare Celeste Berra, Raffaele Marfella, Giuseppe Paolisso, Giuseppe Matarese, Claudio Procaccini, Ilaria Spatocco, Giorgia Mele, Paola de Candia, Antonio Ceriello, Francesco Prattichizzo

**Affiliations:** ^1^ IRCCS MultiMedica Milan Italy; ^2^ Clinic of Laboratory and Precision Medicine IRCCS INRCA Ancona Italy; ^3^ Istituti Clinici Scientifici Maugeri IRCCS Cardiac Rehabilitation Unit of Bari Institute Bari Italy; ^4^ Department of Clinical and Molecular Sciences (DISCLIMO) Università Politecnica delle Marche Ancona Italy; ^5^ Advanced Technology Center For Aging Research IRCCS INRCA Ancona Italy; ^6^ UOC Laboratory of Clinical Medicine With Specialized Areas IRCCS MultiMedica Milan Italy; ^7^ Clinical Microbiology and Microbiome Laboratory Department of Biomedical Sciences For Health University of Milan Milan Italy; ^8^ Department of Endocrinology IRCCS MultiMedica Milan Italy; ^9^ Department of Advanced Medical and Surgical Sciences University of Campania “Luigi Vanvitelli” Naples Italy; ^10^ UniCamillus International Medical University Rome Italy; ^11^ Dipartimento Di Medicina Molecolare e Biotecnologie Mediche Università degli Studi di Napoli Federico II Naples Italy; ^12^ Laboratorio Di Immunologia Istituto Degli Endotipi in Oncologia Metabolismo e Immunologia “G. Salvatore” Consiglio Nazionale delle Ricerche (IEOMI‐CNR) Napoli Italy

**Keywords:** LEP‐R, aging, Ob, inflammation, senescence, SASP, endothelial cells, macrophages, T cells, immunosenescence

## Abstract

Leptin, a proinflammatory adipokine, contributes to the chronic low‐grade inflammation seen in obesity, but its role in age‐related inflammation, that is inflammaging, has been poorly studied. We thus tested the effect of blocking leptin receptor (LEP‐R) binding in five models of inflammaging, that is replicative senescent endothelial cells, hyperglycemia‐treated macrophages or endothelial cells, lipopolysaccharide (LPS)‐exposed macrophages, and TCR‐stimulated CD4+ T cells. In endothelial cells, leptin enhanced senescence, while leptin neutralization suppressed the senescence‐associated secretory phenotype, two effects mediated in part by the mTOR pathway. In both macrophages and endothelial cells, leptin blockade blunted hyperglycemia‐induced inflammation, while in macrophages, it also suppressed the LPS‐induced senescent and proinflammatory phenotype. In T cells, leptin neutralization produced divergent effects, attenuating the expression of inflammatory and senescence markers in conventional T cells but not in regulatory T cells. To assess translational relevance, we measured leptin and inflammaging markers in plasma samples from two independent cohorts, one of healthy individuals (*n* = 121) and another of people with Type 2 diabetes (*n* = 93). In healthy subjects, leptin levels were higher in people with hs‐CRP ≥ 3 mg/dL compared with those below such threshold, also after adjustment for BMI. Leptin levels showed BMI‐adjusted positive correlations with IL‐6 and hs‐CRP in healthy individuals and with hs‐CRP and IL‐8 in people with diabetes. These findings suggest that leptin might fuel inflammaging and that its neutralization exerts anti‐inflammatory effects across multiple, related models.

## Graphical abstract

Multiple mechanisms including senescent cell accumulation, metabolic and endotoxin‐driven stimulation of immune and endothelial cells contribute to inflammaging, the chronic low‐grade inflammatory status underlying age‐related diseases. Blocking leptin signaling in experimental models of inflammaging blunts inflammation in a model‐specific manner while circulating leptin levels are associated with inflammaging markers in humans. These findings identify leptin as a potential contributor to inflammaging.

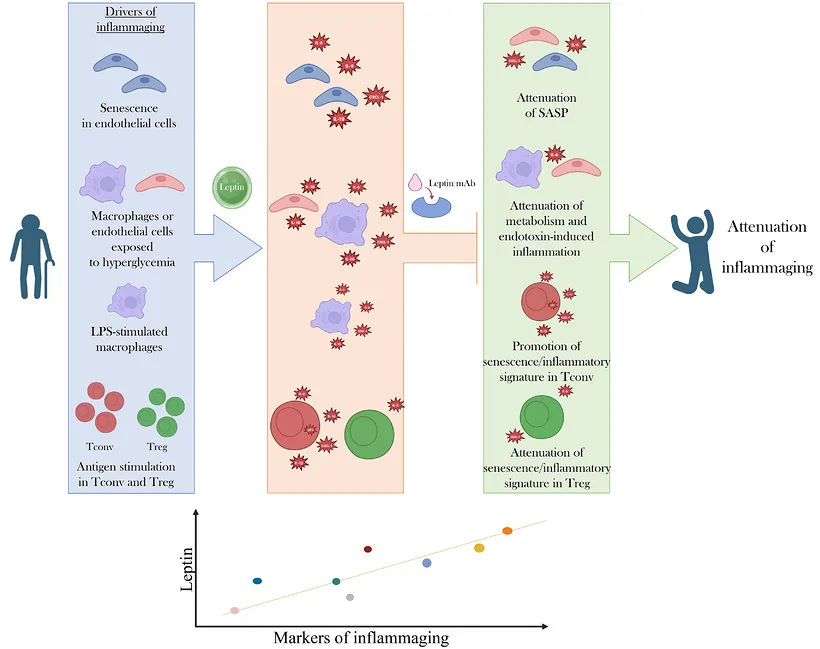

## 1 Introduction

Leptin is a peptide hormone mainly secreted by the adipose tissue [1]. The primary function of leptin is the regulation of hunger, food energy use, and energy balance, through its activity in the central hypothalamus [1, 2]. Leptin levels increase as adipose tissue expands, as a regulatory loop aimed at limiting food intake and blocking the development of overweight [3]. However, most people with obesity develop a condition referred to as leptin resistance and are thus insensitive to the beneficial effects of leptin, also when exogenously administered at therapeutic doses [3, 4]. The resulting hyperleptinemia is held to foster and sustain a chronic inflammatory program in the adipose tissue and at the systemic level in individuals with overweight or obesity [5, 6]. Indeed, leptin has a recognized proinflammatory activity on a range of different cells [6, 7] while observational studies suggest that leptin levels are associated with both systemic inflammation and the development of a range of poor outcomes including cardiovascular diseases [7, 8, 9, 10]. However, despite the vast knowledge relative to the proinflammatory effect of leptin, no study has explored its possible relationship with inflammaging.

Inflammaging is the chronic, sterile, low‐grade inflammation that develops progressively with advancing age and contributes to the development of age‐related diseases [11]. Among other mechanisms, the accumulation of senescent cells and the chronic stimulation of the innate and acquired immune systems by antigens, toxins, and metabolites are believed to represent the major fuels of inflammaging [12]. Mechanistically, the nutrient‐sensing mTOR pathway plays a major role in all these phenomena, regulating the rate of senescence [13], the senescence‐associated secretory phenotype (SASP, i.e., the permanent proinflammatory program of senescent cells) [14, 15], and the response of both innate and acquired immune cells [16, 17]. Considering that leptin affects the mTOR pathway in multiple settings [18], we asked whether leptin neutralization could curb inflammation in models of inflammaging.

To test this hypothesis, we assessed the effect of blocking the activity of leptin on its receptor (LEP‐R) in five widely used in vitro models of inflammaging, that is endothelial cells undergoing replicative senescence [19], macrophages and endothelial cells exposed to hyperglycemia [20, 21], lipopolysaccharide (LPS)‐treated macrophages [21, 22], and T cell receptor (TCR)‐stimulated CD4^+^ T cells [23, 24]. To corroborate the results, we then explored the association between leptin and common markers of inflammaging in plasma samples derived from two distinct cohorts: one of healthy individuals (*n* = 121) and another of people with Type 2 diabetes (pwT2D, *n* = 93), a prototypical age‐related disease characterized by a pervasive proinflammatory status [25, 26].

## 2 Results

### 2.1 Analysis of Leptin and LEP‐R Expression in Senescent Endothelial Cells and Differentiated Macrophages

In order to evaluate the potential relevance of the leptin pathway in our endothelial cell model, we started by assessing the expression of both leptin and LEP‐R in human umbilical vein endothelial cells (HUVEC) in different conditions of senescence. To this aim, we cultured HUVEC until complete growth arrest and evaluated the levels of leptin, LEP‐R, and the most common senescence and SASP markers. As expected, replicative senescent endothelial cells were at least 60% positive for senescence‐associated beta‐galactosidase (SA‐β‐Gal) (Figure 1A,B) and increased the expression of common senescence markers, that is p16 and p21, developing also a fully‐fledged SASP, as shown by the increased abundance of interleukin (*IL*)*‐8* mRNA and of IL‐6 and IL‐8 proteins (Figure 1C–E). LEP‐R was basally expressed in HUVEC and replicative senescence upregulates its expression at the protein, but not mRNA level (Figure 1C–E). On the contrary, HUVEC expressed very low levels of leptin, if compared with MDA‐MB‐231 cells, known to substantially produce and secrete this protein [27] (Figure 1F).

**FIGURE 1:**
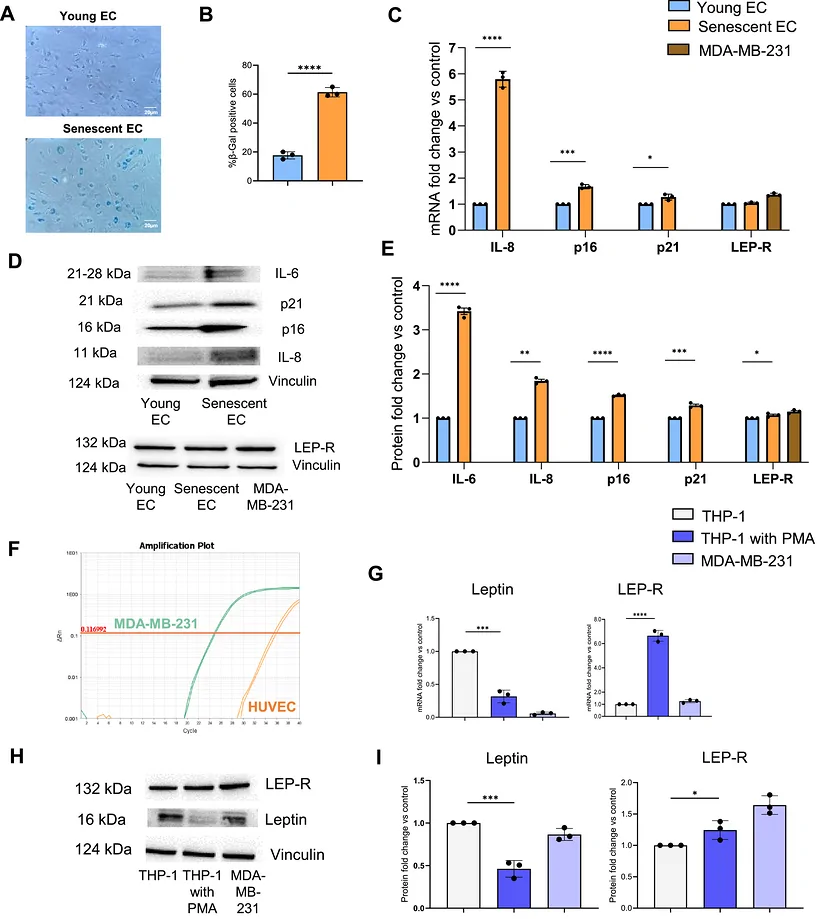
Expression of leptin receptor (LEP‐R) in senescent endothelial cells and differentiated macrophages. (A and B) Representative SA‐β‐Gal staining, along with the relative quantitation, of young and senescent endothelial cells. (C) mRNA expression levels of the senescence markers p16 and p21, of the SASP marker IL‐8, and of LEP‐R presented as fold change in senescent versus young cells. (D and E) Representative Western blots and the relative densitometric analyses of the senescence markers p16 and p21 and of the SASP proteins IL‐6 and IL‐8, as well as of LEP‐R, presented as fold change in senescent versus young cells and normalized using vinculin as internal control. MDA‐MB 231 cells were used as positive control for the expression of leptin receptor for both mRNA and protein assessment. (F) Amplification plots derived from real time PCR of leptin mRNA in endothelial cells (HUVEC), and in MDA‐MB‐231 cell line, used as positive control [27, 28]; the vertical axis is in fluorescent units (ΔRn) while the horizontal axis is in PCR cycle numbers. (G–I) mRNA and protein expression levels of both leptin and LEP‐R in THP‐1 monocytes, induced or not to differentiate into macrophages through PMA treatment. MDA‐MB 231 cells were used as positive control for the expression of both leptin and LEP‐R for mRNA and protein assessment. Data are mean ± SD of three independent experiments. *p < 0.05; **p < 0.01; ***p < 0.001; ****p < 0.0001. For all comparisons, Student's *t*‐test was used. EC: endothelial cells. PMA: phorbol‐12‐myristate‐13‐acetate. LEP‐R: leptin receptor.

We then quantified leptin and LEP‐R in THP‐1 monocytes before and after effective phorbol‐12‐myristate‐13‐acetate (PMA)‐induced differentiation into macrophages (verified through the increased expression of the macrophage markers CD11c, CD301, and CD163; Figure S1). Of note, while the abundance of leptin was reduced in differentiated macrophages, compared with undifferentiated monocytes, the expression of LEP‐R showed the opposite behavior at both the mRNA and the protein levels (Figure 1G–I).

### 2.2 Leptin Treatment Promotes Endothelial Senescence

We thus explored whether the treatment with recombinant leptin (at obesity‐pertinent doses [4, 5]) was able to affect senescence in presenescent (i.e., SA‐β‐Gal < 30%) HUVEC cells (Figure 2A). Continuous treatment for 5 days with multiple doses of recombinant leptin promoted a significant increase in the prevalence of SA‐β‐Gal positive cells and induced the expression of the senescence markers p16 and p21 at both mRNA and protein levels (Figure 2B–D,F,G), with no obvious differences between doses in both cases. The development of senescence was accompanied by a prototypical SASP, as evidenced by the increased expression of the SASP factors plasminogen activator inhibitor (*PAI)‐1, IL‐6, IL‐8*, and *CXCL1* at the mRNA level (Figure 2E), as well as by the increase in the protein levels of IL‐6, IL‐8, and CXCL1 (Figure 2F,G), and of the levels of IL‐6 and MCP‐1 released in the culture media (Figure 2H). This effect was paralleled by the activation of the mTOR pathway, as demonstrated by the increased phosphorylation of mTOR and S6 (Figure 2I,L), an observation that could underlie the prosenescence mechanism of leptin signaling [13].

**FIGURE 2:**
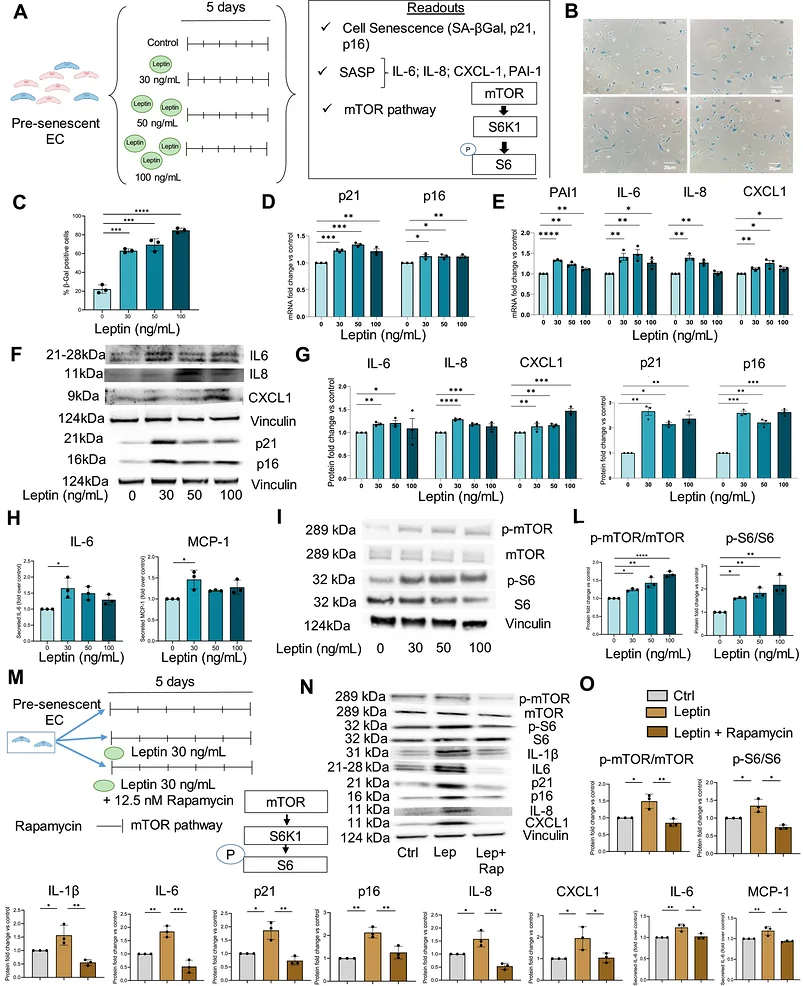
Leptin promotes endothelial senescence. (A) Design of the experiment with the relative readouts. (B and C) Representative SA‐β‐Gal staining, along with the relative quantitation, of presenescent endothelial cells treated with increasing doses of recombinant human leptin (30, 50, and 100 ng/mL) or control (0 ng/mL). (D) mRNA expression levels of the senescence markers p16 and p21 and (E) of the SASP markers PAI‐1, IL‐6, IL‐8, and CXCL1, presented as fold changes over control, in the same experimental settings. (F and G) Representative Western blots and the relative densitometric analyses of IL‐6, IL‐8, CXCL1, p16, and p21 presented as fold changes over control and normalized using vinculin as internal control. (H) Amount of IL‐6 and MCP‐1 released in the cell media in the same experimental conditions, normalized according to cell number and presented as fold over control. (I–L) Representative Western blots and the relative densitometric analyses of p‐mTOR and p‐S6 in the same conditions, normalized according to the respective ratios with mTOR and S6 and also according to vinculin. (M) Design of the rescue experiment with rapamycin. (N and O) Representative Western blots and the relative densitometric analyses of p16, p21, IL‐1β, IL‐6, IL‐8, and CXCL1, presented as fold changes over control and normalized using vinculin as internal control, and of p‐mTOR and p‐S6, normalized according to the respective ratios with mTOR and S6 and also according to vinculin. (P) Amount of IL‐6 and MCP‐1 released in the cell media, normalized according to cell number and presented as fold over control. Data are mean ± SD of three independent experiments. *p < 0.05; **p < 0.01; ***p < 0.001; ****p < 0.0001. For all comparison, ANOVA followed by Holm–Šídák test was used. EC: endothelial cells.

To explore the possible role of mTOR in mediating the observed phenomenon, we exposed presenescent endothelial cells to 30 ng/mL leptin, with or without the presence of rapamycin (Figure 2M), a known mTOR inhibitor [14, 15]. As expected, treatment with rapamycin was able to revert the effect of leptin on the phosphorylation of both mTOR and S6 (Figure 2N,O). Of note, inhibition of mTOR attenuated the development of senescence and the SASP induced by leptin, as demonstrated by the lower abundance of p16, p21, IL‐1β, IL‐6, IL‐8, and CXCL1 at the protein level (Figure 2N,O), and by the decreased secretion of IL‐6 and MCP‐1 in the culture media (Figure 2P).

### 2.3 Leptin Neutralization Suppresses the Endothelial SASP

Based on the above results, we asked whether antagonizing the activity of leptin through the addition of a specific monoclonal antibody (leptin mAb) may attenuate the SASP in senescent endothelial cells, that is with SA‐β‐Gal positivity >60%, grown in a media supplemented with a physiologically pertinent concentration of leptin (Figure 3A). Five days of leptin neutralization was able to reduce the expression of IL‐6, IL‐8, and chemokine C–X–C motif ligand (CXCL)1 at the protein level (Figure 3B,C) and the abundance of IL‐6 and MCP‐1 secreted in the culture media (Figure 3D), without affecting the SASP at the mRNA level (Figure S2). Of note, this effect was observed without changes in the amount of the senescence protein p16 (Figure 3B,C) and of the percentage of SA‐β‐Gal positive cells (Figure S3), and it was accompanied by a decrease in mTOR and S6 phosphorylation (Figure 3E,F), a pathway known to control SASP mRNAs translation [13].

**FIGURE 3:**
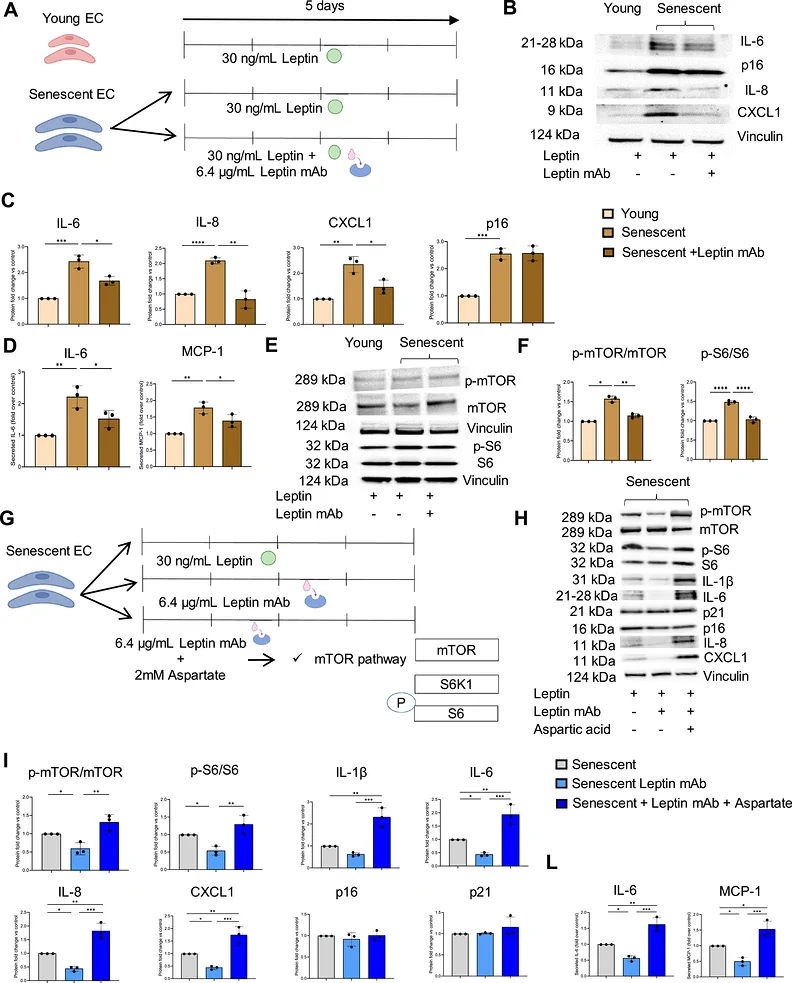
Leptin neutralization attenuates the senescence‐associated secretory phenotype in endothelial cells. (A) Experimental design. (B and C) Representative Western blots and the relative densitometric analyses of IL‐6, p16, IL‐8, and CXCL1, presented as fold changes over control and normalized using vinculin as internal control. (D) Amount of IL‐6 and MCP‐1 released in the cell media, normalized according to cell number and presented as fold over control. (E and F) p‐mTOR and p‐S6 in the same conditions, normalized according to the respective ratios with mTOR and S6 and also to vinculin, in young EC, senescent EC, and senescent EC treated with a leptin antagonist, with all cells grown in the presence of 30 ng/mL of leptin. (G) Design of the rescue experiment with aspartate. (H and I) Representative Western blots and the relative densitometric analyses of p16, p21, IL‐1β, IL‐6, IL‐8, and CXCL1, presented as fold changes over control and normalized using vinculin as internal control, and of p‐mTOR and p‐S6, normalized according to the respective ratios with mTOR and S6 and also according to vinculin. (J) Amount of IL‐6 and MCP‐1 released in the cell media, normalized according to cell number and presented as fold over control. Data are mean ± SD of three independent experiments. *p < 0.05; **p < 0.01; ***p < 0.001; ****p < 0.0001. For all comparison, ANOVA followed by Holm–Šídák test was used. EC: endothelial cells.

Given these findings, we assessed the role of the mTOR pathway in this context and exposed senescent endothelial cells, cultured in the presence of leptin, to the leptin mAb, with or without the cotreatment with 2 mM aspartate (Figure 3G), a demonstrated mTOR activator in endothelial cells [29]. Activation of mTOR promoted the phosphorylation of both mTOR and S6 (Figure 3H,I) and was associated with an increased abundance of the SASP factors IL‐1β, IL‐6, IL‐8, and CXCL1 at the protein level (Figure 3H,I) and with a higher secretion of IL‐6 and MCP‐1 in cell media (Figure 3J), compared with leptin neutralization alone. Of note, senescence rate was not affected, as suggested by the unchanged expression of p16 and p21 (Figure 3H,I).

Overall, these data suggest that leptin promotes senescence in endothelial cells while leptin neutralization attenuates the SASP in senescent endothelial cells supplemented with physiological levels of leptin, two effects ascribable, at least in part, to the modulation of the mTOR pathway.

### 2.4 Leptin Neutralization Attenuates Metabolic Inflammation in Macrophages and Endothelial Cells

Since metabolic dysregulation is known to play a key role in immune cell deranged stimulation in the context of inflammaging [6, 30], we next tested whether leptin neutralization could attenuate the inflammatory response evoked by hyperglycemia in macrophages or endothelial cells cultivated in the presence of leptin (Figure 4A). As hypothesized, treatment with the leptin mAb was able to attenuate the proinflammatory activation promoted by hyperglycemia in macrophages, as evidenced by the lower abundance of *PAI‐1*, *IL‐6*, and *IL‐1α* mRNAs and by the lower protein expression of IL‐6 and IL‐1β in cells treated with the leptin antagonist and compared with hyperglycemia alone (Figure 4B–D).

**FIGURE 4:**
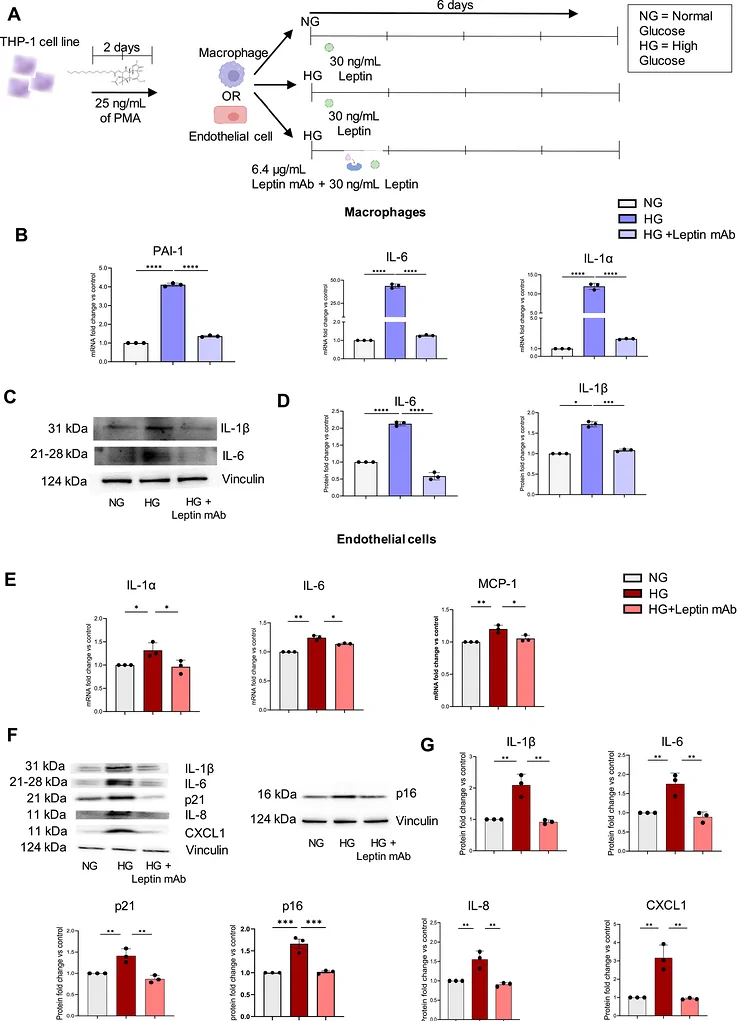
Leptin neutralization attenuates hyperglycemia‐induced inflammation in macrophages. (A) Experimental design. (B) mRNA expression levels of PAI‐1, IL‐6, and IL‐1α, presented as fold changes over control, in macrophages exposed to hyperglycemia with or without leptin antagonist in the presence of leptin supplementation. (C and D) Representative Western blots and the relative densitometric analyses of IL‐6 and IL‐1β, presented as fold changes over control and normalized using vinculin as internal control, in the same experimental settings. (E) mRNA expression levels of IL‐1α, IL‐6, and MCP‐1 mRNAs presented as fold changes over control, in endothelial cells exposed to hyperglycemia with or without leptin antagonist in the presence of leptin supplementation. (F and G) Representative Western blots and the relative densitometric analyses of p16, p21, IL‐1β, IL‐6, IL‐8, and CXCL1, presented as fold changes over control and normalized using vinculin as internal control, in the same experimental settings. Data are mean ± SD of three independent experiments. *p < 0.05; **p < 0.01; ***p < 0.001; ****p < 0.0001. For all comparison, ANOVA followed by Holm–Šídák test was used. NG: normal glucose, that is 10 mM glucose; HG: high glucose, that is 30 mM glucose for macrophages and 25 mM glucose for endothelial cells; PMA: phorbol‐12‐myristate‐13‐acetate.

Similar findings were obtained in endothelial cells. Indeed, hyperglycemia promoted the expression of *IL‐1α, IL‐6*, and *MCP‐1* mRNAs (Figure 4E), and of p16, p21, IL‐1β, IL‐6, IL‐8, and CXCL1 at the protein level (Figure 4F,G), and these effects were attenuated by leptin neutralization (Figure 4E–G), overall suggesting that leptin antagonism ameliorates metabolism‐triggered inflammation.

### 2.5 Leptin Neutralization Attenuates the SASP‐Like Phenotype Induced by LPS in Macrophages

Given the key role of innate immune system stimulation in the development of inflammaging [11], we exposed THP‐1 derived macrophages, cultivated in the presence of leptin, to 1 µg/mL of LPS, a model known to promote the development of a SASP‐like phenotype in this cell‐type [21, 22], with or without the concomitant treatment with the leptin mAb. Treatment with LPS for 24 h induced the accumulation of SA‐β‐Gal, the expression of *IL‐1α, IL‐1β, IL‐6*, and *IL‐8* mRNAs, and an increased abundance of p21, IL‐1β, IL‐6, IL‐8, and CXCL1 at the protein level (Figure S4). Of note, these effects were attenuated by leptin neutralization (Figure S4), suggesting that leptin blockade is able to partially suppress the SASP‐like phenotype induced by LPS in macrophages.

### 2.6 Leptin Neutralization Specifically Modulates CD4^+^ T Cell Responses to TCR Stimulation

The expression of LEP‐R in T cells has been consistently reported [31, 32]. We thus explored the impact of leptin neutralization in TCR‐stimulated primary human CD4^+^ T cells, analyzing the effect on the coding transcriptome separately for proinflammatory conventional T (Tconv) and regulatory T (Treg) cells (Figure 5A), as reported in a previously published study [31]. Stimulation of the TCR complex resulted in a partially divergent effect on the expression of selected inflammatory and senescence genes when comparing Tconv and Treg cells, as evidenced for instance by the selective induction of the senescence gene *p21* only in the former ones (Figure 5B). Treatment with leptin mAb partly attenuated the induction of this signature in Tconv cells while promoting its expression in Treg cells (Figure 5B), highlighting a cell type‐specific effect of leptin neutralization in TCR‐stimulated T cells.

**FIGURE 5:**
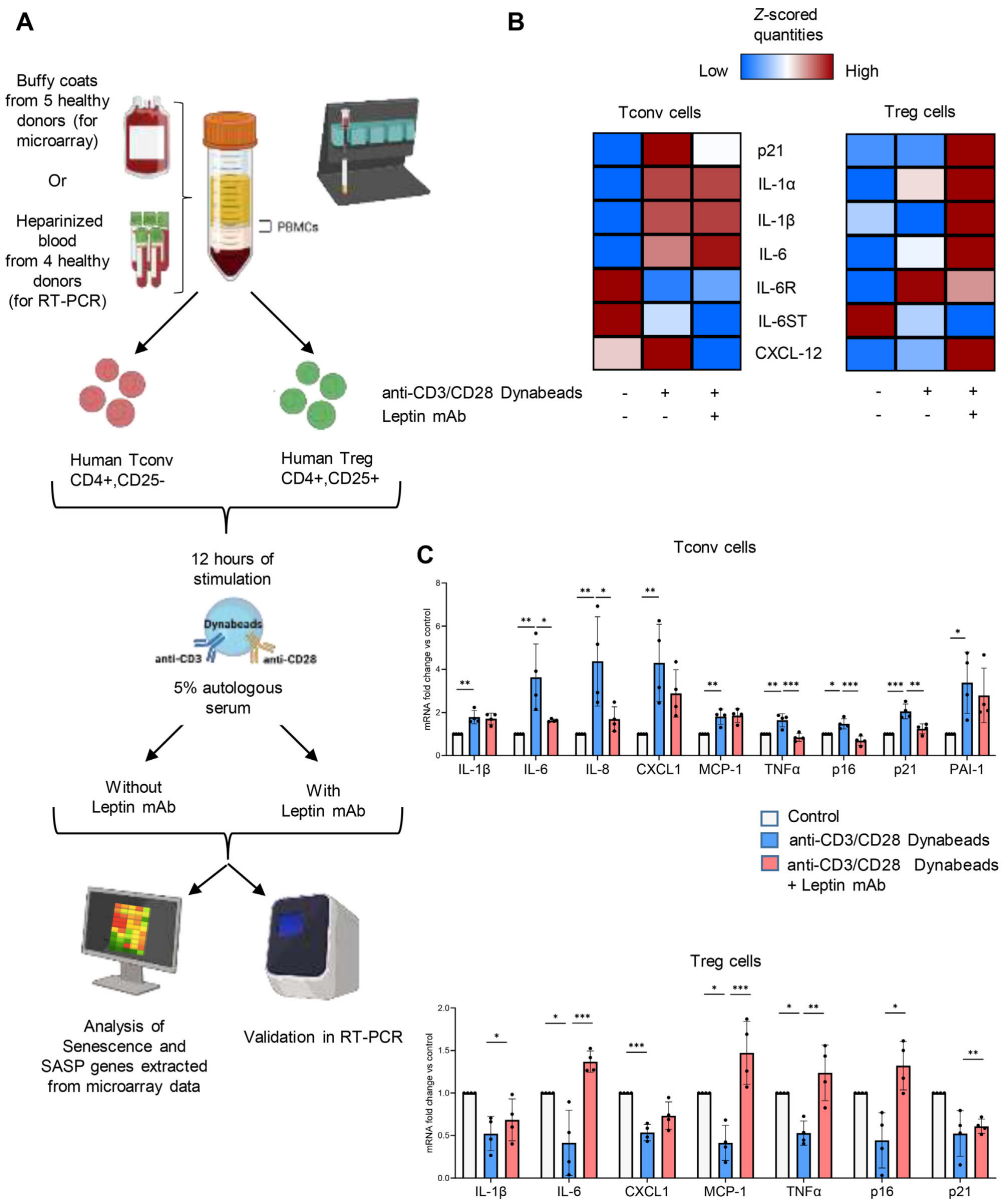
Leptin neutralization differentially modulates the response of antigen‐stimulated Tconv and Treg cells. (A) Experimental design. (B) Heatmap synthesizing the expression of a selected signature of senescence and SASP mRNAs derived from microarray data in Tconv and Treg cells exposed to antigen stimulation with or without the presence of the leptin antagonist. Color codes correspond to row‐scaled *Z*‐scores. (C) Expression of a selected set of senescence and SASP mRNAs assessed through RT‐PCR in Tconv and Treg cells exposed to TCR stimulation for 12 h with or without the presence of the leptin antagonist. Data are mean ± SD of four biological replicates. *p < 0.05; **p < 0.01; ***p < 0.001. For all comparison, ANOVA followed by Holm–Šídák test was used. PBMC: peripheral blood mononuclear cell.

To corroborate microarray data, we replicated the experimental setting and stimulated both primary Treg and Tconv cells with TCR agonists for 12 h in the presence/absence of leptin mAb to assess the expression of senescence and inflammatory mRNAs through RT‐PCR (Figure 5A). Activation of the TCR complex promoted the expression of *p16, p21, IL‐1β, IL‐6, IL‐8, TNFα, MCP‐1, CXCL1*, and *PAI‐1* in Tconv cells, with leptin neutralization attenuating the induction of *p16, p21, IL‐6, IL‐8*, and *TNFα* (Figure 5C). An opposite trend was observed in Treg cells. Indeed, stimulation of the TCR complex was associated with a reduction in the expression of *p16, p21, IL‐1β, IL‐6, TNFα, MCP‐1*, and *CXCL1*, and treatment with the leptin mAb ameliorated the decrease of *p16, IL‐6, TNFα*, and *MCP‐1*. These data sustain the cell type‐specific effects of leptin blockade in the context of stimulation of acquired immunity, with a consistent antisenescence/SASP effect in Tconv cells.

### 2.7 Circulating Leptin Is Associated With Markers of Inflammaging Independently of BMI

In order to gain preliminary insights into the human relevance of these results, we assessed the levels of circulating leptin in a cohort of healthy individuals (*n* = 121), along with the inflammaging markers hs‐CRP, IL‐6, and PAI‐1. Given the prognostic value of hs‐CRP in predicting cardiovascular outcomes, we categorized subjects according to having an hs‐CRP≥ or <3 mg/L, a cut‐off commonly used to distinguish individuals at high risk from those at intermediate or low cardiometabolic risk [33, 34]. The clinical characteristics of the cohort are presented in Table S1. People with hs‐CRP ≥ 3 mg/L, compared with those with hs‐CRP < 3 mg/L, were older and had a higher mean BMI. Given the key role of BMI in determining the levels of both leptin and inflammatory markers [5, 6, 30, 35], and considering that our goal was to explore the association between leptin and inflammaging markers, the subsequent analyses were all adjusted for BMI. Individuals with hs‐CRP ≥ 3 mg/L, compared with those with hs‐CRP < 3 mg/L, had higher mean level of circulating leptin, independently of BMI, that is estimated marginal means 7.36 (95% confidence interval 5.79–8.92) versus 5.01 (95% confidence interval 3.99–6.04); *p* = 0.0157 (Figure 6A). Notably, leptin levels showed a positive correlation with the inflammaging markers hs‐CRP, IL‐6, and PAI‐1, with the first two being significant also after adjustment for BMI (Figure 6B–D). We then explored the relationship of leptin with other clinical variables and observed that its abundance was higher in females compared with males (Figure S5), while no association with age was noted (data not shown).

**FIGURE 6:**
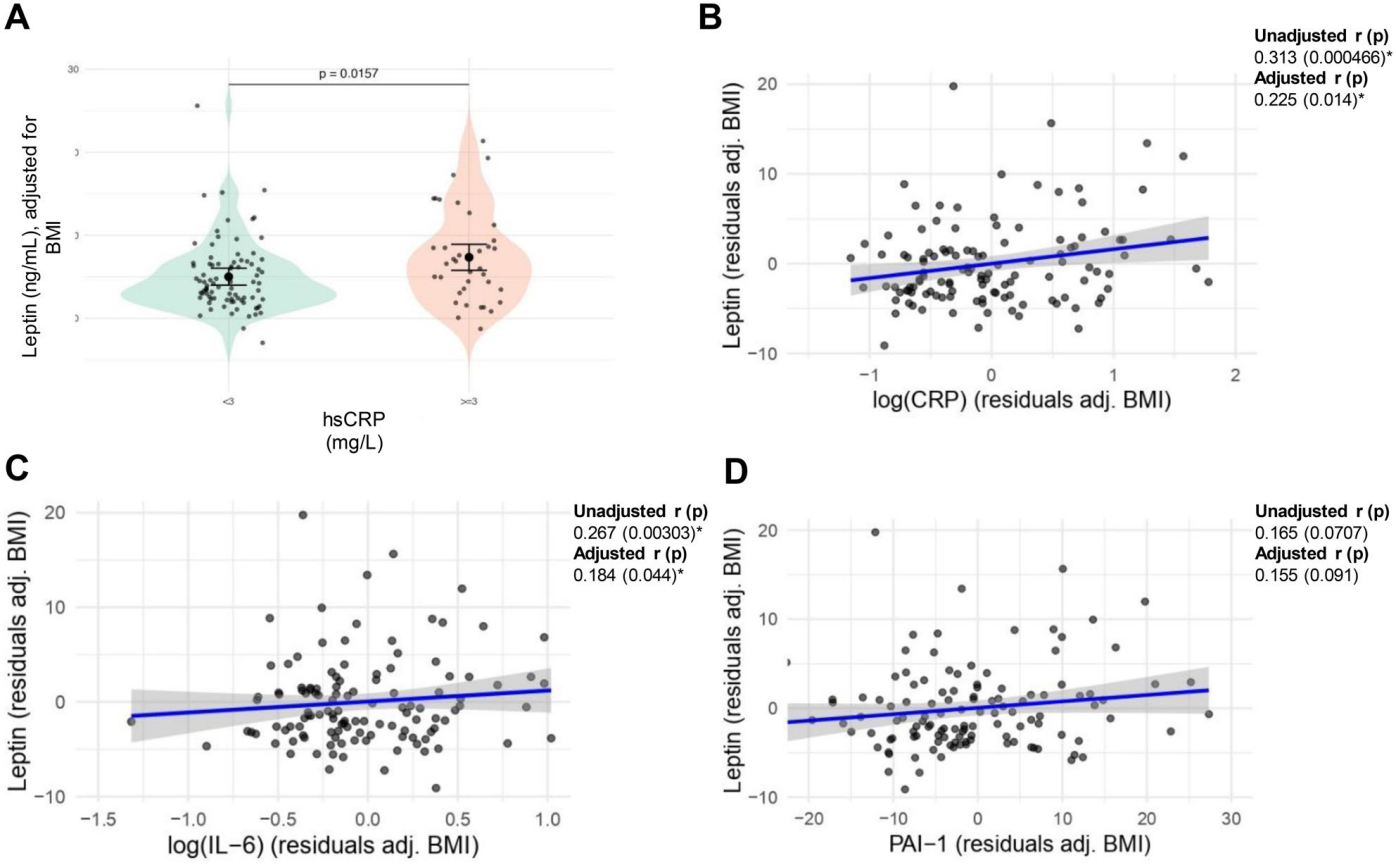
Plasma leptin levels are higher in people with hs‐CRP≥ versus <3 mg/L and correlate with markers of inflammaging in healthy individuals. (A) Levels of plasma leptin in healthy people with hs‐CRP< (*n* = 84) versus ≥3 mg/L (*n* = 37), adjusted for BMI. *p* Value derive from ANCOVA adjusted for BMI. Correlations between plasma leptin levels and hs‐CRP (B), IL‐6 (C), or PAI‐1 (D) in the cohort of 121 healthy individuals. BMI‐adjusted linear regression graphs, Pearson's coefficient, both unadjusted and adjusted for BMI, along with the relative *p* values, are shown. *p < 0.05.

To further substantiate these observations, we dosed leptin and markers of inflammaging in the plasma of 93 pwT2D. The clinical characteristics of the cohort are presented in Table S2. Even in this cohort, we observed a positive correlation between leptin and the inflammaging markers hs‐CRP, IL‐6, and IL‐8, but not with MCP1 (Figure S6). Of note, the correlations between leptin and both hs‐CRP and IL‐8 were significant also after adjustment for BMI (Figure S6). The variable network graph presented in Figure S7 summarizes the relationship between leptin levels and clinically relevant patients’ characteristics and corroborates the findings relative to the higher levels of circulating leptin in females compared with males.

## 3 Discussion

The chronic hyperleptinemia accompanying obesity induces the activation of proinflammatory pathways in a range of tissues [6]. This phenomenon modulates the activity of immune and other cell types, promoting a number of pathogenic mechanisms in a plethora of different conditions, for example, autoimmune and cardiovascular diseases [36, 37]. Our results extend the knowledge relative to the inflammatory effect of leptin, suggesting its role in sustaining inflammaging. Indeed, we have here shown that leptin neutralization by treatment with an antileptin mAb is able to (i) suppress the SASP of replicative senescent endothelial cells, (ii) curb the proinflammatory response generated by hyperglycemia in macrophages and endothelial cells, (iii) ameliorate the SASP‐like phenotype induced by LPS in macrophages, and (iv) differently affect the inflammatory status of TCR‐stimulated CD4^+^ Tconv versus Treg cells. Furthermore, peripheral blood circulating leptin levels are higher in healthy people with increased low‐grade inflammation, independently of BMI. Leptin also showed a positive and BMI‐independent correlation with markers of inflammaging in both healthy individuals and subjects with T2D.

To our knowledge, ours is the first study to specifically explore the impact of leptin neutralization in models of inflammaging. However, previous reports have suggested that leptin may induce a dose‐dependent prosenescence response in chondrocytes and other cell types, even though its specific effect on the development of the SASP was not reported [38, 39]. In the present work, we found that leptin treatment per se induces senescence in endothelial cells and that blocking leptin by an antileptin antibody consistently suppresses the SASP in senescent endothelial cells, even though in some cases, the effect size were modest. Of note, this latter effect was evident at the protein but not mRNA level, an observation possibly ascribable to the suppressing effect of leptin neutralization on the mTOR pathway. Indeed, experiments with mTOR inhibitors and agonists rescued the effect of leptin of endothelial senescence and the SASP, respectively. Consistently, two seminal papers suggested that a fully‐fledged SASP is mTOR dependent and inhibition of this pathway decreases the secretion of inflammatory cytokines by senescent cells, possibly by halting the translation of membrane‐bound IL1α or by differentially regulating the translation of MK2 [14, 15]. Of note, mTOR was also suggested to regulate the rate of senescence itself [13], in line with the results shown here. More broadly, mTOR is held to represent one of the key drivers linking overnutrition and trophic signals to pathways of aging [40]. The observations provided in this manuscript might be placed within this framework, uncovering leptin as an additional, intermediate factor between excess adiposity, and/or nutrition and inflammaging.

The proinflammatory effects of leptin on monocytes, macrophages, and endothelial cells are well established [6, 30]. Similarly, hyperglycemia induces both a transient and a long‐lasting inflammatory response in multiple cell types [19, 41]. Here, we extend such knowledge by showing that inhibiting leptin activity is able to reduce the expression of inflammatory markers induced by glucose excess in both macrophages and endothelial cells, a known model linking overnutrition to low‐grade inflammation [20].

Repeated stimulation of both innate and acquired immunity by both self and non‐self‐molecules is considered one of the key factors sustaining inflammaging [11, 12]. A closely intertwined phenomenon is immunosenescence, that is the gradual deterioration of the immune system observed during age advancement and mainly driven by the continuous challenge of the unavoidable exposure to a variety of antigens [42]. In this context, development of a SASP‐like phenotype by macrophages is held to play a role in both inflammaging and immunosenescence [21]. Here, we demonstrate that leptin neutralization is able to attenuate the prosenescence and proinflammatory response induced by LPS in macrophages, a previously suggested relevant model in this context [21, 22].

Another key event underlying both inflammaging and immunosenescence is the development of dysfunctional T cells, either exhausted or senescent [23, 24]. Our data suggest that leptin neutralization attenuates the induction of senescence markers in Tconv cells but not in Treg cells, along with a partially divergent effect on a selected SASP signature, possibly suggesting a cell‐type‐specific effect in regard to T cell senescence. Of note, leptin neutralization or related strategies aimed at lowering circulating leptin were already suggested as possible approaches to improve the response to specific infectious diseases [43]. However, the hypothesis that halting leptin activity specifically in the elderly may improve their ability to cope with infections deserves future studies.

Blood circulating leptin levels are known to be strictly related to BMI and other measures of adiposity [44]. Fewer studies have explored the impact of age on the amount of plasma leptin levels. Previous research suggests both increased and decreased levels during aging, possibly highlighting a context‐dependent effect [45, 46, 47]. Such discrepancies might be partly attributable to the age range taken into account by the different studies and to the complex remodeling affecting adipose tissue during aging [48]. Indeed, fat mass tends to accumulate during aging but then declines in the oldest old [48]. Our cohorts have a relatively limited age range and thus we did not observe a clear trend of leptin levels during aging. As a result, we were unable to provide a definitive answer on this point, hence additional, larger studies, including the youngest young and the oldest old, are needed to establish circulating leptin's trend during aging. In addition, fat redistribution is also observed during aging with an increase in visceral fat coupled by a decrease in subcutaneous fat [49]. To this respect, a study in rats suggested that adipose tissue infiltration in skeletal muscle is associated with an increased expression of leptin, which in turn promotes muscle inflammation in aged animals [50]. Independently of the tissue and systemic modulation of leptin during aging, the data relative to the correlations between leptin and inflammatory markers provided here and relative to leptin levels in people with high versus low hs‐CRP, both observed also after adjustment for BMI support the hypothesis that leptin may sustain inflammaging, and suggest the exploration of leptin antagonists as a strategy to ameliorate such phenomenon.

At present, only leptin itself and one leptin analogue, that is metreleptin, have reached clinical practice while leptin antagonists are not currently indicated for any condition. In particular, metreleptin is used in people with generalized lipodystrophy, either congenital or acquired, a condition characterized by a pathological redistribution of adipose tissue coupled with a severe leptin deficiency [51]. However, metreleptin shows no efficacy in decreasing body weight in people with obesity and without lipodystrophy, likely due to the phenomenon of leptin resistance [51, 52]. The potential efficacy of antagonizing leptin has already been suggested for a variety of diseases. In particular, it has been proposed as a therapeutic strategy to block leptin‐dependent activation of autoreactive responses or tumor progression [53, 54]. However, most of the leptin antagonists promote hyperphagia and are thus intrinsically linked to unwanted weight gain [55]. Recent approaches are trying to uncouple the metabolic from the immune effects of leptin [56], possibly allowing the use of such drugs without inducing weight gain. On the other hand, leptin signaling is already indirectly targeted by dietetic approaches. Indeed, a consistent decrease in the blood circulating levels of leptin accompanies caloric restriction, both short and long term [57]. Acute starvation induces a drop of leptin independently of adiposity while long‐term dietetic interventions promote a decrease in leptin levels that parallels weight loss [57]. Of note, caloric restriction is one of the most well‐established antiaging interventions [58], which suggests that leptin reduction may be one of the many intermediate beneficial mediators of such an approach against inflammaging and, more broadly, aging.

In summary, here we show that leptin neutralization blunts inflammatory responses across five pertinent models of inflammaging, while peripheral blood circulating leptin levels correlate with markers of inflammaging, being more abundant in healthy people with increased low‐grade inflammation, independently of BMI. These findings suggest that leptin may fuel inflammaging and propose the investigation of peripheral leptin antagonists or other strategies aimed at lowering leptin levels as promising approaches to mitigate this condition.

## 4 Materials and Methods

### 4.1 Cell Cultures, Reagents, and Treatments

Human monocytic THP‐1 cells (ATCC) were cultured in RPMI‐1640 medium supplemented with 10% heat‐inactivated fetal bovine serum (FBS), 1% penicillin/streptomycin, and 1% l‐glutamine (all from Euroclone). HUVEC from pooled donors (Clonetics, Lonza) were cultivated in endothelial basal medium (EBM‐2, CC‐3156; Lonza) supplemented with SingleQuot Bullet Kit (CC‐4176; Lonza). The cells were seeded at a density of 5000/cm^2^ in T75 flasks and sub‐cultured until complete growth arrest for the induction of replicative senescence, which was verified through SA‐β‐Gal staining (#9860; Cell Signaling). Cells were considered senescent when at least 60% positive for SA‐β‐Gal. MDA‐MB 231 (ATCC) were cultured with RPMI 1640 supplemented with 10% FBS and 1% penicillin/streptomycin. These cells were not used in any experiments but served as a positive control for the expression of both leptin and LEP‐R, as suggested in previous literature [27, 28].

In one set of experiments, presenescent endothelial cells (i.e., SA‐β‐Gal < 30%) were exposed to escalating doses (30, 50, and 100 ng/mL) of recombinant leptin (L4146; Merck), concentrations pertinent to those observed in overweight or obese subjects [9, 59], for 5 days, with daily media change and leptin supplementation. In another set of experiments, senescent endothelial cells (i.e., SA‐β‐Gal > 50%) were treated for 5 days with 6.4 µg/mL of the mouse monoclonal neutralizing Ab against human leptin (leptin mAb, MAB398; R&D Systems) in the presence of 30 ng/mL of recombinant leptin, in line with the suggested neutralization range [60].

Markers of senescence and the SASP to be assessed were selected according to existing guidelines [61, 62].

To evaluate the role of the mTOR pathways in the development of the observed phenotypes, two sets of experiments were performed. In one set, presenescent endothelial cells were treated with 30 ng/mL of recombinant leptin for 5 days, with or without the presence of 12.5 nM rapamycin (553210; Sigma–Aldrich), an mTOR inhibitor [14]. In the other set, senescent endothelial cells were treated for 5 days with 6.4 µg/mL of the leptin mAb in the presence of 30 ng/mL leptin, with or without the presence of 2 mM aspartate (A6558; Sigma–Aldrich), an mTOR activator [29].

For the experiments with hyperglycemia‐induced inflammation, THP‐1 monocytes were differentiated into M0 macrophages with 25 ng/mL PMA (P8139‐10MG; Merck) for 2 days and then exposed to 30 mM glucose in a media supplemented with 30 ng/mL of recombinant leptin for 6 days, with or without leptin neutralization obtained through treatment with 6.4 µg/mL of the leptin antagonist. The same experimental design was applied to endothelial cells, with the exception that no pretreatment was applied and 25 mM of glucose, instead of 30 mM, was used to mimic hyperglycemia.

For the induction of the SASP‐like phenotype in macrophages, THP‐1 derived macrophages (differentiated as described above) were treated with LPS (1 µg/mL, L6529; Sigma) for 24 h [22] in the presence of 30 ng/mL of leptin and with or without the cotreatment of 6.4 µg/mL of the leptin antagonist.

### 4.2 RNA Extraction and RT qPCR

Total RNA was extracted using the RNA Purification Kit (37500; Norgen Biotek) and assessed for concentration and purity with a NanoDrop spectrophotometer (Thermo Fisher). Samples with a 260/280 ratio of approximately 2.0 were selected for further analysis. One microgram of RNA was reverse‐transcribed using the Superscript III Reverse Transcriptase Kit (Invitrogen) following the manufacturer's protocol. Quantitative real‐time PCR (qRT‐PCR) was conducted on a QuantStudio 6 Flex detection system (Applied Biosystems) with TB Green Premix Ex Taq reagents (RR420A; Takara Bio). GAPDH was used as the reference gene for normalization in all the presented analyses. The thermal cycling conditions followed previously published protocols [63] and the primers used are listed in Table S3.

### 4.3 SA‐Beta Galactosidase Assay

Cellular senescence was evaluated using the Senescence β‐Galactosidase Staining Kit (#9860; Cell Signalling) according to the manufacturer's instructions. Briefly, cells were washed with PBS, fixed for 10–15 min at room temperature, and incubated overnight at 37°C in a dry incubator without CO_2_ using freshly prepared β‐galactosidase staining solution at pH 6.0. Senescent cells were identified by the development of blue staining and analyzed under a light microscope at 200× magnification. The percentage of positive cells was determined by counting at least 500 cells/well by two independent researchers. The mean count for each condition was used as one replicate.

### 4.4 Western Blot

Cells were lysed in RIPA buffer (R0278; Merck) with 10% protease inhibitor (11873580001 cOmplete, EDTA‐free Protease Inhibitor Cocktail; Merck). Protein concentration was determined using the Bradford assay (23200; Thermo Fisher). For Western blots, 50 µg of lysate was separated by electrophoresis using PAGE gels (NP0335BOX, NuPAGE Bis‐Tris Mini Protein Gels, 4–12%; Thermo Fisher) and transferred to nitrocellulose membranes (GE10600020, 0.45 µm; Merck). After blocking with 5% nonfat dried milk (70166‐500G; Merck) or 5% bovine serum albumin (A1470‐100G; Merck), membranes were incubated overnight at 4°C with the following primary antibodies: anti‐IL‐8 (ab110727; Abcam), anti‐IL‐6 (#12153; Cell Signalling), anti‐CXCL1/CXCL2 (#24376; Cell Signalling), anti‐IL‐1β (ab254360; Abcam), anti‐p16 (#92803; Cell Signalling), anti‐p21 (#2947; Cell Signalling), anti‐phospho(p)‐S6 (#4858; Cell Signalling), anti‐S6 (#2217; Cell Signalling), anti‐p‐mTOR (#5536; Cell Signalling), anti‐mTOR (#2983; Cell Signalling), antileptin (PAI‐052; ThermoFisher), antileptin receptor (ab282571; Abcam), and antivinculin (#13901; Cell Signalling) all diluted 1:1000. Secondary IgG HP‐conjugated antirabbit HRP‐linked antibody (#7074; Cell Signalling, 1:3000) were applied for 1 h at room temperature. Immunoreactive proteins were revealed with WESTAR ETA ULTRA C 2.0 (XLS075,0100; Cyanagen) using UVITEC Alliance Q9. Vinculin was used as the loading control. Densitometric analysis was performed with UVITEC Nine Alliance.

### 4.5 Assessment of Secreted IL‐6 and MCP‐1 in Conditioned media

At the end of the experiments, cells were counted for normalization purposes, and the supernatants were collected, centrifuged to remove cell debris, and stored at −80°C. To measure the abundance of cytokines released in the culture media, the amounts of IL‐6 (BMS213HS; Invitrogen) and MCP‐1 (BMS281; Invitrogen) were assessed through ELISA kits, according to the manufacturer's instructions. Cell number was used to normalize data, which are presented as fold over control.

### 4.6 Transcriptomics and RT‐PCR of TCR‐Stimulated T Cells

Transcriptomics data were retrieved from a publicly available microarray dataset deposited in GEO (accession number: GSE154401) [31], reporting the expression profile of human CD4^+^CD25^+^ Treg and CD4^+^CD25^−^ Tconv cells, isolated from peripheral blood mononuclear cells (PBMCs) and TCR‐stimulated in vitro in the presence or absence of an antileptin antibody (leptin mAb). From the whole normalized microarray dataset available in the relative publication [31], only data regarding specific transcripts (senescence and SASP‐related ones) were extrapolated and heatmaps with the relative, row‐scaled, *z*‐score values were built by using the mean of each experimental group. Genes were selected based on existing guidelines suggesting their reliability as markers of senescence and the SASP [61, 62], and when consistently expressed, that is being detected in all the replicates assessed.

To corroborate microarray data, we replicated this experimental setting to assess senescence and inflammatory mRNAs through RT‐PCR in TCR‐stimulated Treg and Tconv primary cells. PBMCs were isolated from 20 mL of heparinized blood trough Ficoll‐Paque (Sigma–Aldrich) according to manufacturer's protocol. Blood samples were obtained from four healthy donors, two males and two females (mean age 36.3 ± 4.8 years, mean BMI 23.8 ± 1.6). From PBMCs, CD4+CD25+ Treg (positive selection) and CD4+CD25− Tconv (negative selection) cells were isolated through Dynabeads Regulatory CD4+/CD25+ T Cell Kit (Invitrogen) according to manufacturer's instructions. Both primary cell types were stimulated with Dynabeads Human T‐Activator CD3/CD28 beads (0.2 beads per cell; Invitrogen) in the presence of 5% autologous serum, with or without the presence of 20 µg/mL of the leptin mAb for 12 h. Markers of senescence and inflammation were thus assessed through RT‐PCR as described above. Among all the mRNAs tested, only those consistently expressed (i.e., cycle threshold < 35) in the four subjects and in all the conditions tested were included in the analysis. For this reason, *IL‐1α* was excluded from the analysis relative to both Tconv and Treg cells while *IL‐8* and *PAI‐1* were excluded only from the analysis relative to Treg cells.

### 4.7 Sample Size Calculation

Hs‐CRP is the most commonly used marker to assess low‐grade inflammation in multiple contexts including aging [64]; hence, we set the correlation between leptin and hs‐CRP as the endpoint to calculate the needed sample size. Previous studies suggest a correlation between these two variables in people with obesity or other metabolism‐related conditions, with all correlations showing *r*‐values > 0.3 [65, 66]. Thus, setting a *r* = 0.3 and assuming an α (two‐tailed) = 0.05, a *β* = 0.2, and assuming also that the distribution of the variables involved was normal, it results that 85 people are needed to explore a possible significant correlation between leptin and inflammaging markers with the intention of using a Pearson correlation.

### 4.8 Study Cohort 1: Healthy Individuals

Samples were retrieved from a previously published cohort of control subjects recruited among nonconsanguineous relatives of outpatients attending the Metabolic Diseases and Diabetology Department of IRCCS INRCA (27437767) [67]. All subjects underwent a medical evaluation that included a complete history and clinical examination. Individuals with a clinical history of coronary artery disease, stroke, and peripheral vascular diseases were excluded from the study. Subjects with either a positive history of diabetes mellitus or a fasting blood glucose ≥126 mg/dL after confirmation on repeat testing were considered people with T2D and excluded. People with hs‐CRP > 10 mg/L were considered to have an ongoing acute inflammatory condition, as suggested by guidelines [33, 34], and were thus excluded. The study was approved by the Institutional Review Board of IRCCS INRCA hospital (Approval No. 34/CdB/03). Written informed consent was obtained from each participant in accordance with the principles of the Declaration of Helsinki.

### 4.9 Study Cohort 2: People With Type 2 Diabetes

Plasma samples derive from a cross‐sectional cohort of 100 pwT2D, previously recruited and characterized at the Diabetology Unit of IRCCS MultiMedica between September 2020 and April 2021 [63]. Inclusion criteria were: (i) being older than 18 years, (ii) having received a diagnosis of T2D, and (iii) being on a stable therapy, that is no new drug prescription during the last 6 months. Exclusion criteria were: (i) having received a diagnosis of diabetes different from T2D, (ii) having any of the following conditions, that is pregnancy, short life expectancy, chronic or acute inflammatory status (cancer, autoimmune diseases, etc.), and (iii) having hs‐CRP > 10 mg/L. Information relative to anthropometric factors, medical history, therapies, and the presence/absence of diabetes complications was also obtained. Seven individuals were excluded due to hemolytic samples (*n* = 2) or for having hs‐CRP > 10 mg/L (*n* = 5), yielding a final cohort of 93 subjects. The local ethics committee (IRCCS MultiMedica, Milan, Italy, protocol n.MM335.2018) approved the original protocol and all participants provided their written informed consent.

### 4.10 Laboratory Variables and ELISAs

Blood samples were collected after overnight fasting. Laboratory variables were measured by standard procedures. One EDTA blood tube was centrifuged at 2000×*g* for 15 min at 4°C to obtain EDTA plasma samples, immediately stored at −80°C. EDTA plasma samples were then used to measure leptin (EIA‐2395, DRG), IL‐6 (BMS213HS; Invitrogen), IL‐8 (KHC0084; Invitrogen), PAI‐1 (BMS2033; Invitrogen), and MCP‐1 (BMS281; Invitrogen) with ELISA kits, according to the manufacturer's instructions.

### 4.11 Statistical Analysis

For in vitro experiments, we used *t*‐test to compare the effect of treatments between two groups while for more than two conditions we performed the ANOVA test followed by the Holm–Šídák test. For the cohorts’ data, normality was assessed through the Shapiro–Wilk test. Continuous variables are reported as median (interquartile range) while categorical variables are reported as n and percentage. People in the cohort of healthy individuals (Cohort 1) were categorized according to hs‐CRP≥ or <3 mg/L, a commonly used cut‐off to identify people at high cardiometabolic risk [33, 34]. Baseline characteristics were compared between the two groups with Mann–Whitney *U* test for continuous variables and Chi‐squared test for categorical variables. Since body weight is a key determinant of both circulating leptin levels and systemic low‐grade inflammation [35], we compared the levels of leptin in the two groups through ANCOVA adjusted for BMI. For the same reason, to explore the possible correlations between leptin and inflammaging markers, we tested Pearson's correlation adjusted for BMI, which were performed also in Cohort 2 (people with diabetes). For this purpose, IL‐6 and hs‐CRP were log‐transformed to facilitate data visualization and reduce skewness. Statistical analyses were carried out using Prism 10 (GraphPad) for in vitro data and with either First Look Data or R (version 4.1) for cohort data. Statistical significance was defined as a *p* value of <0.05.

## Supporting Information

Additional supporting information can be found online in the Supporting Information section.

## Author Contributions

A.C. and F.P. conceived the idea and wrote the manuscript. V.P., R.L.G., A.M., F.C., and C.F. planned and performed most of the experiments, analyzed data, and prepared figures. P.d.C, I.S., and G.Mel. analyzed data relative to T cells. A.G., J.S., G.Mat., C.P., P.d.C., F.O., F.R., L.D., C.C.B., R.M., and G.P. provided background expertise, managed the human cohort or samples, and/or critically reviewed the manuscript. All authors have read and approved the final manuscript.

## Funding

This work has been supported by the Italian Ministry of Health—Ricerca Corrente to IRCCS MultiMedica and through starting grant (SG‐2021‐12372752) to R.L.G.

## Ethics Statement

The study with healthy individuals was approved by the Institutional Review Board of IRCCS INRCA hospital (Approval No. 34/CdB/03) while that with people with diabetes was approved by the local ethics committee (IRCCS MultiMedica, Milan, Italy, protocol n.MM335.2018). All participants in both cohorts provided their written informed consent.

## Conflicts of Interest

The authors declare no conflicts of interest.

## Supporting information




**Supporting File**: Mco271082‐sup‐0001‐SuppMat.pdf.

## Data Availability

The raw array data for the CD4^+^ T cell experiments are available in the open GEO repository [https://www.ncbi.nlm.nih.gov/geo/query/acc.cgi?acc = GSE154401] (GSE154401). The anonymized datasets relative to the levels of leptin and other markers in humans are available upon request through email to the corresponding author (F.P.). Materials used are all commercially available and reported in *Materials and Methods* section.

## References

[1] Y. Zhang , R. Proenca , M. Maffei , et al., “Positional Cloning of the Mouse Obese Gene and Its Human Homologue,” Nature 372 (1994): 425–432. 10.1038/372425a0 7984236

[2] C. S. Mantzoros , “The Role of Leptin in Human Obesity and Disease: a Review of Current Evidence,” Annals of Internal Medicine 130 (1999): 671–680. 10.7326/0003-4819-130-8-199904200-00014 10215564

[3] M. G. Myers, Jr , R. L. Leibel , R. J. Seeley , et al., “Obesity and Leptin Resistance: Distinguishing Cause from Effect,” Trends in Endocrinology and Metabolism 21 (2010): 643–651. 10.1016/j.tem.2010.08.002 PMC2967652 20846876

[4] H. S. Moon , G. Matarese , A. M. Brennan , et al., “Efficacy of Metreleptin in Obese Patients with Type 2 Diabetes: Cellular and Molecular Pathways Underlying Leptin Tolerance,” Diabetes 60 (2011): 1647–1656. 10.2337/db10-1791 PMC3114380 21617185

[5] G. Dessie , B. Ayelign , Y. Akalu , et al., “Effect of Leptin on Chronic Inflammatory Disorders: Insights to Therapeutic Target to Prevent Further Cardiovascular Complication,” Diabetes, Metabolic Syndrome and Obesity 14 (2021): 3307–3322. 10.2147/DMSO.S321311 PMC8296717 34305402

[6] A. Perez‐Perez , F. Sanchez‐Jimenez , T. Vilarino‐Garcia , et al., “Role of Leptin in Inflammation and Vice Versa,” International Journal of Molecular Sciences 21 no. 16 (2020):5887. 10.3390/ijms21165887 PMC7460646 32824322

[7] V. P. Puurunen , A. Kiviniemi , S. Lepojarvi , et al., “Leptin Predicts Short‐Term Major Adverse Cardiac Events in Patients with Coronary Artery Disease,” Annals of Medicine 49 (2017): 448–454. 10.1080/07853890.2017.1301678 28300429

[8] O. Mayer, Jr , J. Bruthans , J. Seidlerova , et al., “High Leptin Status Indicates an Increased Risk of Mortality and Heart Failure in Stable Coronary Artery Disease,” Nutrition, Metabolism, and Cardiovascular Diseases 32 (2022): 2137–2146. 10.1016/j.numecd.2022.06.006 35843790

[9] S. Zhao , C. M. Kusminski , and P. E. Scherer , “Adiponectin, Leptin and Cardiovascular Disorders,” Circulation Research 128 (2021): 136–149. 10.1161/CIRCRESAHA.120.314458 PMC7799441 33411633

[10] S. Soderberg , B. Ahren , J. H. Jansson , et al., “Leptin Is Associated with Increased Risk of Myocardial Infarction,” Journal of Internal Medicine 246 (1999): 409–418. 10.1046/j.1365-2796.1999.00571.x 10583712

[11] C. Franceschi , M. Bonafe , S. Valensin , et al., “Inflamm‐aging. An Evolutionary Perspective on Immunosenescence,” Annals of the New York Academy of Sciences 908 (2000): 244–254. 10.1111/j.1749-6632.2000.tb06651.x 10911963

[12] C. Franceschi , P. Garagnani , P. Parini , et al., “Inflammaging: A New Immune‐Metabolic Viewpoint for Age‐Related Diseases,” Nature Reviews Endocrinology 14 (2018): 576–590. 10.1038/s41574-018-0059-4 30046148

[13] Z. N. Demidenko , S. G. Zubova , E. I. Bukreeva , et al., “Rapamycin Decelerates Cellular Senescence,” Cell Cycle 8 (2009): 1888–1895. 10.4161/cc.8.12.8606 19471117

[14] R. M. Laberge , Y. Sun , A. V. Orjalo , et al., “MTOR Regulates the Pro‐Tumorigenic Senescence‐Associated Secretory Phenotype by Promoting IL1A Translation,” Nature Cell Biology 17 (2015): 1049–1061. 10.1038/ncb3195 PMC4691706 26147250

[15] N. Herranz , S. Gallage , M. Mellone , et al., “mTOR Regulates MAPKAPK2 Translation to Control the Senescence‐Associated Secretory Phenotype,” Nature Cell Biology 17 (2015): 1205–1217. 10.1038/ncb3225 PMC4589897 26280535

[16] S. L. Collins , M. H. Oh , I. H. Sun , et al., “mTORC1 Signaling Regulates Proinflammatory Macrophage Function and Metabolism,” Journal of Immunology 207 (2021): 913–922. 10.4049/jimmunol.2100230 34290107

[17] C. Procaccini , V. De Rosa , M. Galgani , et al., “An Oscillatory Switch in mTOR Kinase Activity Sets Regulatory T Cell Responsiveness,” Immunity 33 (2010): 929–941. 10.1016/j.immuni.2010.11.024 PMC3133602 21145759

[18] L. Dempsey , “Leptin and mTor,” Nature Immunology 13 (2012): 939.

[19] F. Prattichizzo , V. De Nigris , E. Mancuso , et al., “Short‐term Sustained Hyperglycaemia Fosters an Archetypal Senescence‐Associated Secretory Phenotype in Endothelial Cells and Macrophages,” Redox Biology 15 (2018): 170–181. 10.1016/j.redox.2017.12.001 PMC5735298 29253812

[20] G. Matacchione , J. Perugini , E. Di Mercurio , et al., “Senescent Macrophages in the Human Adipose Tissue as a Source of Inflammaging,” Geroscience 44 (2022): 1941–1960. 10.1007/s11357-022-00536-0 PMC9616990 35247131

[21] F. Prattichizzo , M. Bonafe , F. Olivieri , et al., “Senescence Associated Macrophages and ”Macroph‐Aging“: Are They Pieces of the Same Puzzle?,” Aging (Albany NY) 8 (2016): 3159–3160. 10.18632/aging.101133 PMC5270660 27941213

[22] H. Wang , H. Fu , R. Zhu , et al., “BRD4 Contributes to LPS‐Induced Macrophage Senescence and Promotes Progression of Atherosclerosis‐Associated Lipid Uptake,” Aging (Albany NY) 12 (2020): 9240–9259. 10.18632/aging.103200 PMC7288959 32392533

[23] W. G.‐G. F. Santiago‐Cruz , “T Lymphocytes in Aging: CD38 as a Novel Contributor Between Inflammaging and Immunosenescence,” Journal of Cellular Immunology 6, no. 4 (2024): 148–162.

[24] W. Xu and A. Larbi , “Markers of T Cell Senescence in Humans,” International Journal of Molecular Sciences 18 no. 8 (2017):1742. 10.3390/ijms18081742 PMC5578132 28796199

[25] F. Prattichizzo , A. Giuliani , J. Sabbatinelli , et al., “Prevalence of Residual Inflammatory Risk and Associated Clinical Variables in Patients with Type 2 Diabetes,” Diabetes, Obesity and Metabolism 22 (2020): 1696–1700. 10.1111/dom.14081 32394604

[26] F. Prattichizzo , P. de Candia , and A. Ceriello , “Diabetes and Kidney Disease: Emphasis on Treatment with SGLT‐2 Inhibitors and GLP‐1 Receptor Agonists,” Metabolism 120 (2021): 154799. 10.1016/j.metabol.2021.154799 34029597

[27] L. Otvos Jr , I. Kovalszky , M. Riolfi , et al., “Efficacy of a Leptin Receptor Antagonist Peptide in a Mouse Model of Triple‐Negative Breast Cancer,” European Journal of Cancer 47 (2011): 1578–1584. 10.1016/j.ejca.2011.01.018 21353530

[28] R. L. Linares , J. G. S. Benitez , M. O. Reynoso , et al., “Modulation of the Leptin Receptors Expression in Breast Cancer Cell Lines Exposed to Leptin and Tamoxifen,” Scientific Reports 9 (2019): 19189. 10.1038/s41598-019-55674-x PMC7222035 31844100

[29] R. E. Oberkersch , G. Pontarin , M. Astone , et al., “Aspartate Metabolism in Endothelial Cells Activates the mTORC1 Pathway to Initiate Translation During Angiogenesis,” Developmental Cell 57 (2022): 1241–1256, e1248. 10.1016/j.devcel.2022.04.018 35580611

[30] A. H. Berg and P. E. Scherer , “Adipose Tissue, Inflammation, and Cardiovascular Disease,” Circulation Research 96 (2005): 939–949. 10.1161/01.RES.0000163635.62927.34 15890981

[31] C. Procaccini , S. Garavelli , F. Carbone , et al., “Signals of Pseudo‐Starvation Unveil the Amino Acid Transporter SLC7A11 as Key Determinant in the Control of Treg Cell Proliferative Potential,” Immunity 54 (2021): 1543–1560, e1546. 10.1016/j.immuni.2021.04.014 34004141

[32] G. M. Lord , G. Matarese , J. K. Howard , et al., “Leptin Modulates the T‐Cell Immune Response and Reverses Starvation‐Induced Immunosuppression,” Nature 394 (1998): 897–901. 10.1038/29795 9732873

[33] W. L. Roberts and A. Cdc , “CDC/AHA Workshop on Markers of Inflammation and Cardiovascular Disease: Application to Clinical and Public Health Practice: Laboratory Tests Available to Assess Inflammation–Performance and Standardization: A Background Paper,” Circulation 110 (2004): e572–576. 10.1161/01.CIR.0000148986.52696.07 15611384

[34] P. M. Ridker , “Clinical Application of C‐Reactive Protein for Cardiovascular Disease Detection and Prevention,” Circulation 107 (2003): 363–369. 10.1161/01.cir.0000053730.47739.3c 12551853

[35] V. Pellegrini , R. La Grotta , F. Carreras , et al., “Inflammatory Trajectory of Type 2 Diabetes: Novel Opportunities for Early and Late Treatment,” Cells 13 no. 19 (2024):1662. 10.3390/cells13191662 PMC11476093 39404426

[36] P. de Candia , F. Prattichizzo , S. Garavelli , et al., “The Pleiotropic Roles of Leptin in Metabolism, Immunity, and Cancer,” Journal of Experimental Medicine 218 no. 5 (2021):e20191593. 10.1084/jem.20191593 PMC8056770 33857282

[37] M. S. Poetsch , A. Strano , and K. Guan , “Role of Leptin in Cardiovascular Diseases,” Frontiers in Endocrinology (Lausanne) 11 (2020): 354. 10.3389/fendo.2020.00354 PMC7325922 32655492

[38] X. Zhao , P. Huang , G. Li , et al., “Activation of the Leptin Pathway by High Expression of the Long Form of the Leptin Receptor (Ob‐Rb) Accelerates Chondrocyte Senescence in Osteoarthritis,” Bone & Joint Research 8 (2019): 425–436. 10.1302/2046-3758.89.BJR-2018-0325.R2 PMC6775539 31588359

[39] H. Chen , B. Shi , X. Feng , et al., “Leptin and Neutrophil‐Activating Peptide 2 Promote Mesenchymal Stem Cell Senescence Through Activation of the Phosphatidylinositol 3‐Kinase/Akt Pathway in Patients with Systemic Lupus Erythematosus,” Arthritis & Rheumatology 67 (2015): 2383–2393. 10.1002/art.39196 PMC12230990 25989537

[40] M. V. Blagosklonny , “TOR‐Driven Aging: Speeding Car Without Brakes,” Cell Cycle 8 (2009): 4055–4059. 10.4161/cc.8.24.10310 19923900

[41] F. Miao , X. Wu , L. Zhang , et al., “Genome‐Wide Analysis of Histone Lysine Methylation Variations Caused by Diabetic Conditions in Human Monocytes,” Journal of Biological Chemistry 282 (2007): 13854–13863. 10.1074/jbc.M609446200 17339327

[42] Z. Liu , Q. Liang , Y. Ren , et al., “Immunosenescence: Molecular Mechanisms and Diseases,” Signal Transduction and Targeted Therapy 8 (2023): 200. 10.1038/s41392-023-01451-2 PMC10182360 37179335

[43] C. Palma , C. La Rocca , V. Gigantino , et al., “Caloric Restriction Promotes Immunometabolic Reprogramming Leading to Protection from Tuberculosis,” Cell Metabolism 33 (2021): 300–318, e312. 10.1016/j.cmet.2020.12.016 33421383

[44] J. M. Friedman , “Leptin and the Endocrine Control of Energy Balance,” Nature Metabolism 1 (2019): 754–764. 10.1038/s42255-019-0095-y 32694767

[45] A. M. Isidori , F. Strollo , M. More , et al., “Leptin and Aging: Correlation with Endocrine Changes in Male and Female Healthy Adult Populations of Different Body Weights,” Journal of Clinical Endocrinology & Metabolism 85 (2000): 1954–1962. 10.1210/jcem.85.5.6572 10843181

[46] G. Paolisso , S. Ammendola , A. Del Buono , et al., “Serum Levels of Insulin‐Like Growth Factor‐I (IGF‐I) and IGF‐Binding Protein‐3 in Healthy Centenarians: Relationship with Plasma Leptin and Lipid Concentrations, Insulin Action, and Cognitive Function,” Journal of Clinical Endocrinology & Metabolism 82 (1997): 2204–2209. 10.1210/jcem.82.7.4087 9215295

[47] Y. Arai , K. Kamide , and N. Hirose , “Adipokines and Aging: Findings from Centenarians and the Very Old,” Frontiers in Endocrinology (Lausanne) 10 (2019): 142. 10.3389/fendo.2019.00142 PMC6426744 30923512

[48] M. Y. Ou , H. Zhang , P. C. Tan , et al., “Adipose Tissue Aging: Mechanisms and Therapeutic Implications,” Cell Death and Disease 13 (2022): 300. 10.1038/s41419-022-04752-6 PMC8980023 35379822

[49] J. L. Kuk , T. J. Saunders , L. E. Davidson , et al., “Age‐related Changes in Total and Regional Fat Distribution,” Ageing Research Reviews 8 (2009): 339–348. 10.1016/j.arr.2009.06.001 19576300

[50] R. Tazawa , K. Uchida , H. Fujimaki , et al., “Elevated Leptin Levels Induce Inflammation through IL‐6 in Skeletal Muscle of Aged Female Rats,” BMC Musculoskeletal Disorders 20 (2019): 199. 10.1186/s12891-019-2581-5 PMC6511122 31077169

[51] K. Chou and C. M. Perry , “Metreleptin: First Global Approval,” Drugs 73 (2013): 989–997. 10.1007/s40265-013-0074-7 23740412

[52] S. B. Heymsfield , A. S. Greenberg , K. Fujioka , et al., “Recombinant Leptin for Weight Loss in Obese and Lean Adults: a Randomized, Controlled, Dose‐Escalation Trial,” JAMA 282 (1999): 1568–1575. 10.1001/jama.282.16.1568 10546697

[53] L. Zabeau , F. Peelman , and J. Tavernier , “Antagonizing Leptin: Current Status and Future Directions,” Biological Chemistry 395 (2014): 499–514. 10.1515/hsz-2013-0283 24523306

[54] Y. Yu , Y. Liu , F. D. Shi , et al., “Cutting Edge: Leptin‐Induced RORgammat Expression in CD4+ T Cells Promotes Th17 Responses in Systemic Lupus Erythematosus,” Journal of Immunology 190 (2013): 3054–3058. 10.4049/jimmunol.1203275 PMC3608794 23447682

[55] A. Gertler , “Development of Leptin Antagonists and Their Potential Use in Experimental Biology and Medicine,” Trends in Endocrinology and Metabolism 17 (2006): 372–378. 10.1016/j.tem.2006.09.006 17010639

[56] L. Zabeau , J. Wauman , J. Dam , et al., “A Novel Leptin Receptor Antagonist Uncouples Leptin's Metabolic and Immune Functions,” Cellular and Molecular Life Sciences 76 (2019): 1201–1214. 10.1007/s00018-019-03004-9 PMC11105424 30659329

[57] K. Mendoza‐Herrera , A. A. Florio , M. Moore , et al., “The Leptin System and Diet: A Mini Review of the Current Evidence,” Frontiers in Endocrinology (Lausanne) 12 (2021): 749050. 10.3389/fendo.2021.749050 PMC8651558 34899599

[58] A. Di Francesco , A. G. Deighan , L. Litichevskiy , et al., “Dietary Restriction Impacts Health and Lifespan of Genetically Diverse Mice,” Nature 634 (2024): 684–692. 10.1038/s41586-024-08026-3 PMC11485257 39385029

[59] J. Beltowski , “Leptin and Atherosclerosis,” Atherosclerosis 189 (2006): 47–60. 10.1016/j.atherosclerosis.2006.03.003 16580676

[60] https://resources.rndsystems.com/pdfs/datasheets/mab398.pdf?v=20250227.

[61] E. Gonzalez‐Gualda , A. G. Baker , L. Fruk , et al., “A Guide to Assessing Cellular Senescence in Vitro and in Vivo,” The FEBS Journal 288 (2021): 56–80. 10.1111/febs.15570 32961620

[62] M. Ogrodnik , J. Carlos Acosta , P. D. Adams , et al., “Guidelines for Minimal Information on Cellular Senescence Experimentation in Vivo,” Cell 187 (2024): 4150–4175. 10.1016/j.cell.2024.05.059 PMC11790242 39121846

[63] R. La Grotta , P. de Candia , F. Olivieri , et al., “Anti‐inflammatory Effect of SGLT‐2 Inhibitors via Uric Acid and Insulin,” Cellular and Molecular Life Sciences 79 (2022): 273. 10.1007/s00018-022-04289-z PMC9064844 35503137

[64] J. N. Justice , L. Ferrucci , A. B. Newman , et al., “A Framework for Selection of Blood‐Based Biomarkers for Geroscience‐Guided Clinical Trials: Report from the TAME Biomarkers Workgroup,” Geroscience 40 (2018): 419–436. 10.1007/s11357-018-0042-y PMC6294728 30151729

[65] C. Vales‐Villamarin , O. de Dios , I. Perez‐Nadador , et al., “Leptin Concentrations Determine the Association Between High‐Sensitivity C‐Reactive Protein Levels and Body Mass Index in Prepubertal Children,” Nutrients 15 no. 10 (2023):2388. 10.3390/nu15102388 PMC10221072 37242271

[66] P. M. António Pires , A. M. Pereira , and J. Marinho , “Pro‐Inflammatory Triggers in Childhood Obesity: Correlation Between Leptin, Adiponectin and High‐Sensitivity C‐reactive Protein in a Group of Obese Portuguese Children,” Revista Portuguesa de Cardiologia 33, no. 11 (2014): 691–697. 10.1016/j.repc.2014.04.004 25448795

[67] F. Olivieri , M. Bonafe , L. Spazzafumo , et al., “Age‐ and Glycemia‐Related miR‐126‐3p Levels in Plasma and Endothelial Cells,” Aging (Albany NY) 6 (2014): 771–787. 10.18632/aging.100693 PMC4221921 25324472

