# Supplementary material for: Leptin Neutralization Attenuates Inflammaging in Endothelial and Immune Cell Models
Source: MedComm (2020). 2026 Oct 23;7(11):e71082. doi: 10.1002/mco2.71082 (PMC13693919; doi:10.1002/mco2.71082)
Supplement: Supplementary file 1 [file MCO2-7-e71082-s001.pdf]

Supplementary Material

Supplement to: Pellegrini V., *et al.* Leptin Neutralization Attenuates Inflammaging in Endothelial and Immune Cell Models

TABLE OF CONTENTS

1. **Table S1**. Clinical characteristics of the cohort of healthy individuals, categorized according to having a hs-CRP ≥ or < 3 mg/L……………..……………………………………..…..1
2. **Table S2.** Clinical characteristics of the cohort of people with diabetes................................2
3. **Table S3**. List of the primers' sequences used…………....…………..................................3
4. **Figure S1**. THP-1 differentiation into macrophages……………...…….…………………...4
5. **Figure S2**. Leptin neutralization does not regulate the SASP at the mRNA level in endothelial cells…………………………………………………………………………5
6. **Figure S3**. Leptin neutralization does not modulate the prevalence of SA-βGal positivity of senescent endothelial cells..…………………………………………..................................6
7. **Figure S4**. Leptin neutralization attenuates the SASP-like phenotype induced by LPS in macrophages..…..……………………...7
8. **Figure S5**. Leptin levels in healthy females and males…....…...…………………………….9
9. **Figure S6**. Association between leptin and markers of inflammaging in people with type 2 diabetes………..…………………………………….………......………………………10
10. **Figure S7**. Variable network graph of correlations between multiple variables in people with type 2 diabetes……………………..……………………….……………………….11
11. **Uncropped Western Blot**……………………………………………………………….12

Table S1. Clinical characteristics of the study cohort 1 (healthy individuals), categorized according to hs-CRP strata.

Variable[1]	hs-CRP<3<br>N = 84	hs-CRP ≥3<br>N = 37	p-value[2]
**Age (years)**	57 [42, 64]	67 [56, 78]	**<0.001**
**Sex (males)**	41 (49%)	11 (30%)	0.079
**Body mass index (kg/m²)**	25.8 [23.4, 28.1]	27.5 [25.5, 29.8]	**0.005**
**Total cholesterol (mg/dL)**	211 [181, 234]	221 [194, 236]	0.4
**HDL cholesterol (mg/dL)**	57 [48, 71]	52 [44, 59]	0.074
**non-HDL cholesterol (mg/dL)**	154 [121, 176]	166 [137, 181]	0.2
**LDL cholesterol (mg/dL)**	121 [100, 141]	134 [106, 145]	0.3
**Fasting glucose (mg/dL)**	94 [85, 99]	95 [91, 104]	**0.017**
**AST (U/L)**	19.0 [16.0, 24.0]	21.0 [18.0, 24.0]	0.4
**ALT (U/L)**	35 [31, 41]	35 [32, 43]	0.6
**Waist-to-hip ratio**	0.87 [0.81, 0.95]	0.91 [0.85, 0.96]	0.086
**Urea (mg/dL)**	37 [32, 43]	35 [32, 44]	0.8
**eGFR (mL/min/1.73m²)**	87 [71, 99]	78 [70, 88]	0.061
**Creatinine (mg/dL)**	0.90 [0.70, 1.00]	0.80 [0.70, 0.90]	0.8
**Uric acid (mg/dL)**	4.60 [3.75, 5.35]	4.80 [4.00, 5.50]	0.4
**White blood cells (10³/µL)**	6.12 [4.87, 7.30]	6.51 [5.70, 7.21]	0.13
**Hemoglobin (g/dL)**	14.20 [13.30, 14.95]	14.00 [13.50, 14.90]	0.5
**Platelets (10³/µL)**	235 [193, 268]	231 [183, 269]	>0.9
**Hypertension**	24 (29%)	18 (49%)	0.054
**Lipid-lowering therapy**	8 (9.5%)	3 (8.1%)	>0.9
**Antihypertensive therapy**	24 (29%)	17 (46%)	0.10
**PAI-1 (ng/mL)**	17 [12, 23]	21 [16, 31]	**0.013**
**IL-6 (pg/mL)**	1.21 [0.86, 1.70]	2.83 [2.03, 4.13]	**<0.001**

[1]Median [Q1, Q3] or n (%); [2]Significant differences, according to Mann-Whitney U test or Chi-Squared test, are highlighted in bold.

Table S2. Clinical characteristics of the study cohort 2 (people with diabetes).

**Variable**	**People with T2D (n = 93)**
**Age (years)**	66 (58-72)
**Male, n (%)**	51 (54.8)
**BMI (Kg/m2)**	29.2 (26.6-32.4)
**Previous CVD, n (%)**	19 (20.4)
**Diabetes duration (years)**	10 (5-16)
**eGFR (ml/min)**	58.5 (70.2-92.1)
**HbA1c, n (%)**	6.52 (6.1-6.9)
**hs-CRP (mg/dl)**	1.3 (0.5-4.6)
**Total cholesterol (mg/dl)**	163 (142-193)
**LDL cholesterol (mg/dl)**	96 (78-113)
**HDL cholesterol (mg/dl)**	46 (39-55)
**Triglycerides (mg/dl)**	120 (97-153)

Main clinical characteristics of the cohort of 93 people with type 2 diabetes enrolled in the study. Continuous variables are presented as median (Q1-Q3), while categorical variables are presented as number (n) and the relative percentage (%).

Abbreviations for both tables:

CVD: cardiovascular diseases; HbA1c: glycated hemoglobin; HDL: high density lipoprotein; LDL: low density lipoprotein; eGFR: estimated glomerular filtration rate; hs-CRP: high sensitivity C-reactive protein; BMI: body mass index; AST: aspartate transaminase; ALT: alanine aminotransferase.

Table S3. **List of the primers' sequences used in the study.**

**Primer**	**Sequence**
CD11c for	TGA CAT TGC ATC GAA GCC CTC
CD11c rev	TCC GTA CCC TCA ATG GCA AAG
CD163 for	ACT GCA AGA ACT GGC AAT GG
CD163 rev	CCA TGC TTC ACT TCA ACA CG
CD301 for	AGC TGA GGT GGA GGG TTT
CD301 rev	GTG TGC CTT CTG CGT AGT
CXCL-1 for	TCC TGC ATC CCC CAT AGT TA
CXCL-1 rev	CTT CAG GAA CAG CCA CCA GT
GAPDH for	TCC TCT GAC TTC AAC AGC GAC
GAPDH rev	TCT CTC TTC CTC TTG TGC TCT T
IL-1α for	CTT TCC CTG CCT GAC CTT ATT
IL-1α rev	GAA TGA AGC TAC TGC CCT ACT C
IL-6 for	TGC AAT AAC CAC CCC TGA CC
IL-6 rev	GTG CCC ATG CTA CAT TTG CC
IL-8 for	GGA CAA GAG CCA GGA AGA AA
IL-8 rev	CCT ACA ACA GAC CCA CAC AAT A
Leptin for	GCT GTG CCC ATC CAA AAA GTC C
Leptin rev	CCC AGG AAT GAA GTC CAA ACC G
Leptin Receptor for	CCA AAA TTC AGG TCC TCT CAT AGG
Leptin Receptor rev	GCA GTC TAT GCT GTT CAG GTG C
p16 for	CAT AGA TGC CGC GGA AGG T
p16 rev	CTA AGT TTC CCG AGG TTT CTC AGA
p21 for	ACC GAG ACA CCA CTG GAG GG
p21 rev	CCT GCC TCC TCC CAA CTC ATC
PAI-1 for	CAG ATG AGG ACA GAG TGG TTT C
PAI-1 rev	GTG GGC TGA TTA GGC TTC TAT T

Figure S1.

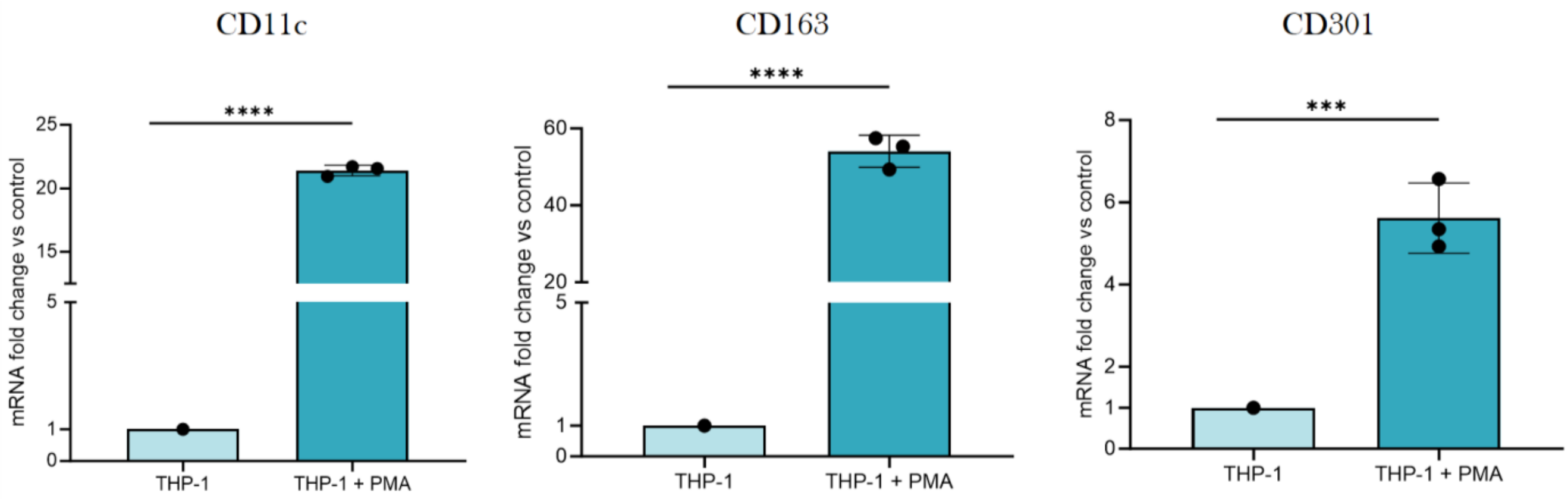

THP-1 monocytes differentiation into macrophages. mRNA expression of the macrophage markers CD11c, CD163, and CD301 in untreated THP-1 monocytes and THP-1 differentiated into macrophages through treatment with 25 ng/mL PMA for 2 days. Data are mean ± SD of three independent experiments. ***$p < 0.001$; ****$p<0.0001$ Student's t test. PMA: Phorbol-12-myristate-13-acetate.

Figure S2.

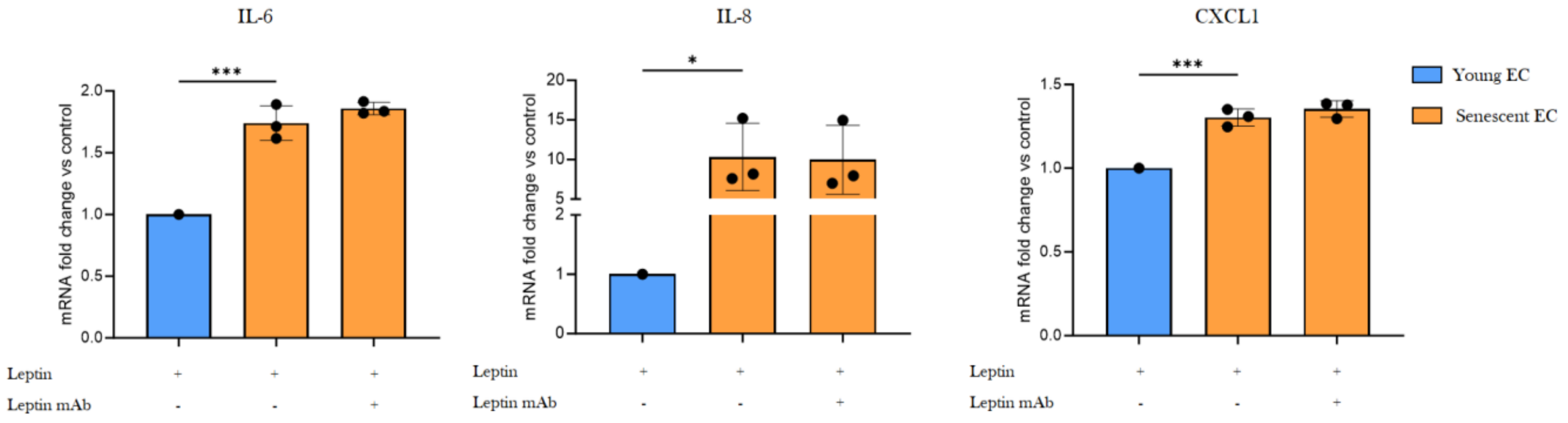

Leptin neutralization does not regulate the SASP at the mRNA level. Expression of the senescence-associated secretory phenotype (SASP) mRNAs IL-6, IL-8, and CXCL-1, in young (blue histograms) and senescent (orange) endothelial cells, treated or not with the leptin mAb. Data are mean ± SD of three independent experiments. *$p < 0.05$; **$p < 0.01$; ***$p < 0.001$. For all comparison, ANOVA followed by Holm-Šídák test was used. EC= endothelial cells.

Figure S3.

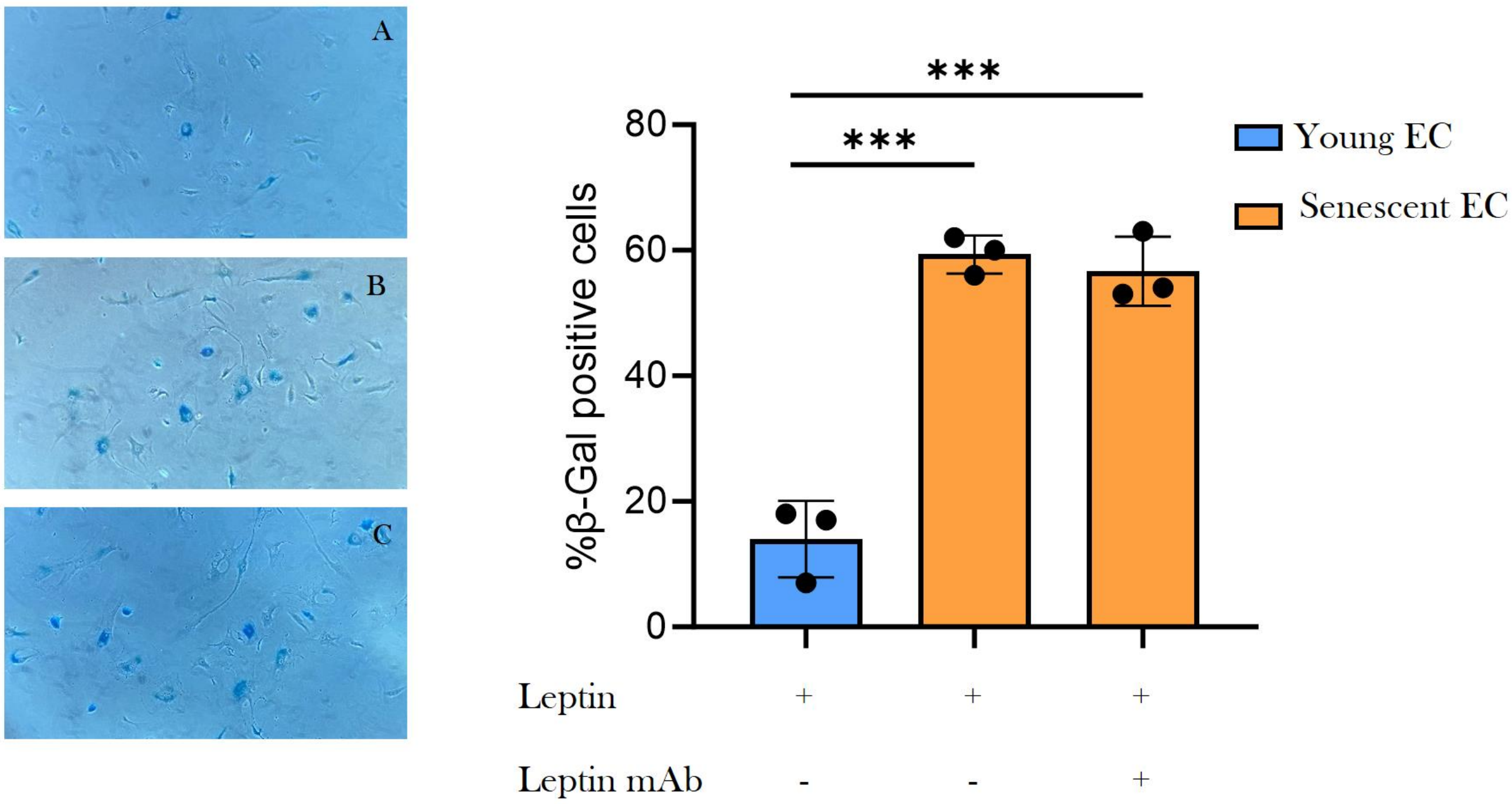

Leptin neutralization does not modulate the prevalence of SA-βGal positivity of senescent endothelial cells. Representative SA-βGal staining, along with the relative quantitation, of young and senescent endothelial cells, treated or not with the leptin mAb. Data are mean ± SD of three independent experiments. ***$p < 0.001$; ANOVA followed by Holm-Šídák. EC= endothelial cells.

Figure S4.

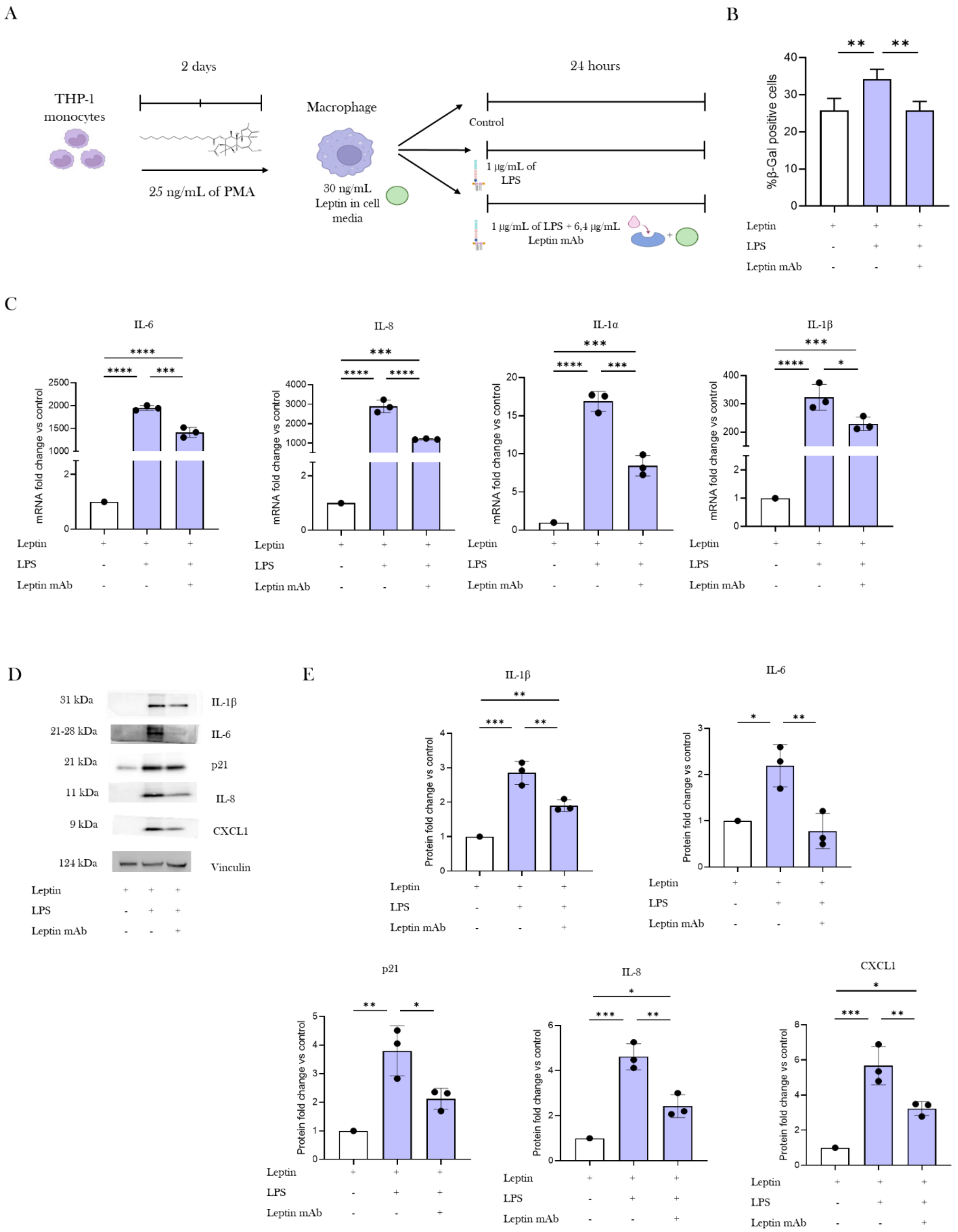
A
THP-1
monocytes
2 days
25 ng/mL of PMA
Macrophage
30 ng/mL
Leptin in cell
media
24 hours
Control
1 µg/mL of
LPS
1 µg/mL of LPS + 6,4 µg/mL
Leptin mAb
B
%β-Gal positive cells
C
IL-6
IL-8
IL-1α
IL-1β
mRNA fold change vs control
D
31 kDa
IL-1β
21-28 kDa
IL-6
21 kDa
p21
11 kDa
IL-8
9 kDa
CXCL1
124 kDa
Vinculin
E
IL-1β
IL-6
p21
IL-8
CXCL1
Protein fold change vs control
Leptin
LPS
Leptin mAb

Leptin neutralization attenuates the SASP-like phenotype induced by LPS in macrophages. A Graphical summary of the experimental design. B Quantitation of SA-βGal positive macrophages, treated or not with the leptin mAb. C Expression of IL-1α, IL-1β, IL-6, and IL-8 mRNAs in the same experimental setting. D,E Representative Western Blots and the relative densitometric analyses of p21, IL-1β, IL-6, IL-8, and CXCL1, presented as fold changes over control and normalized using Vinculin as internal control. Data are mean ± SD of three independent experiments. *$p < 0.05$; **$p < 0.01$; ***$p < 0.001$. For all comparison, ANOVA followed by Holm-Šídák test was used. THP-1= monocytes; PMA=Phorbol 12-myristate 13-acetate.

Figure S5.

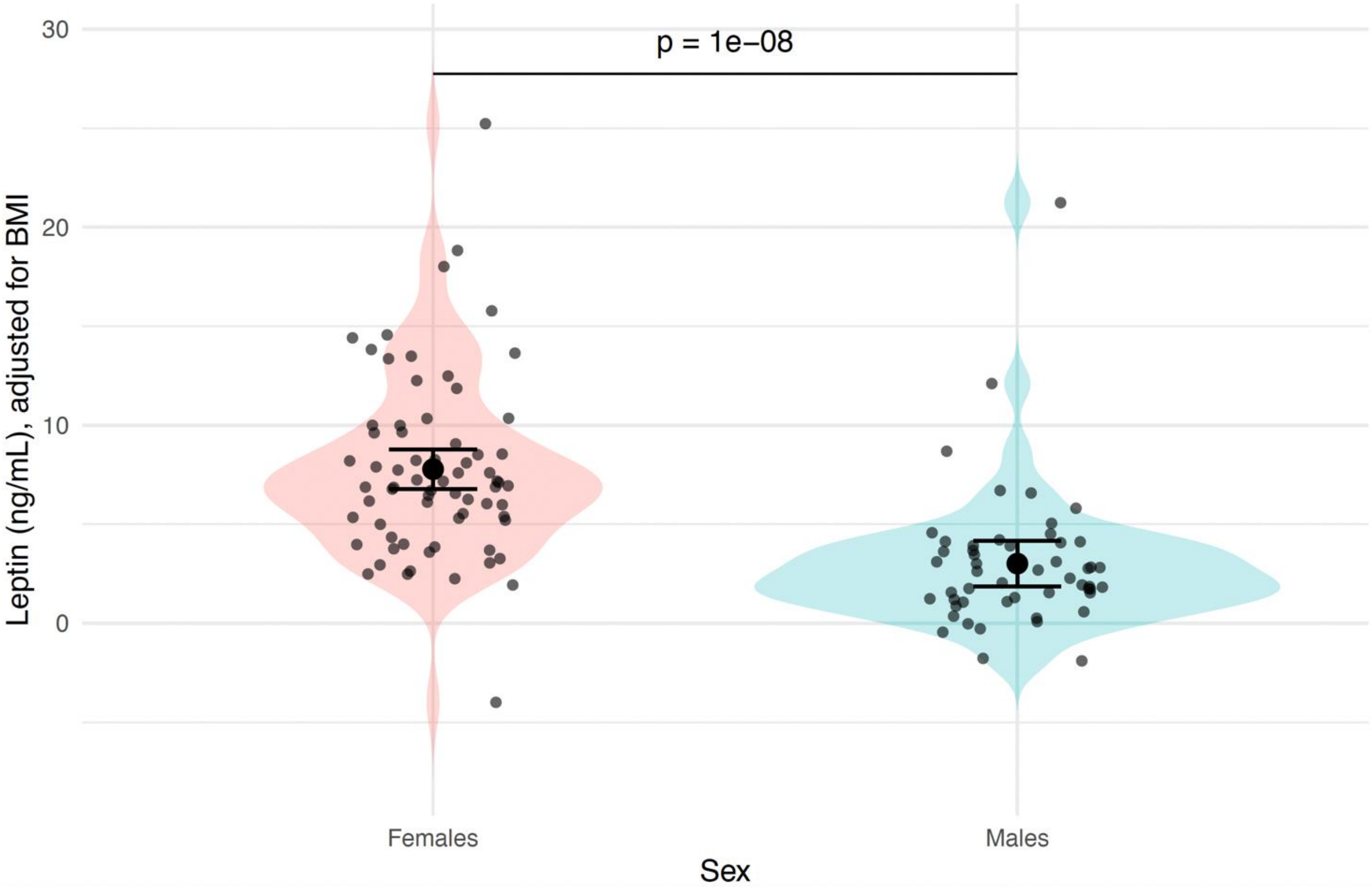

Sex-stratified leptin levels in the cohort of healthy individuals. Violin plots, along with individual points, showing the BMI-adjusted levels of plasma leptin in females (pink) and males (light blue). Estimated marginal means ±95% confidence intervals are shown. The reported p value derives from ANCOVA adjusted for BMI.

Figure S6.

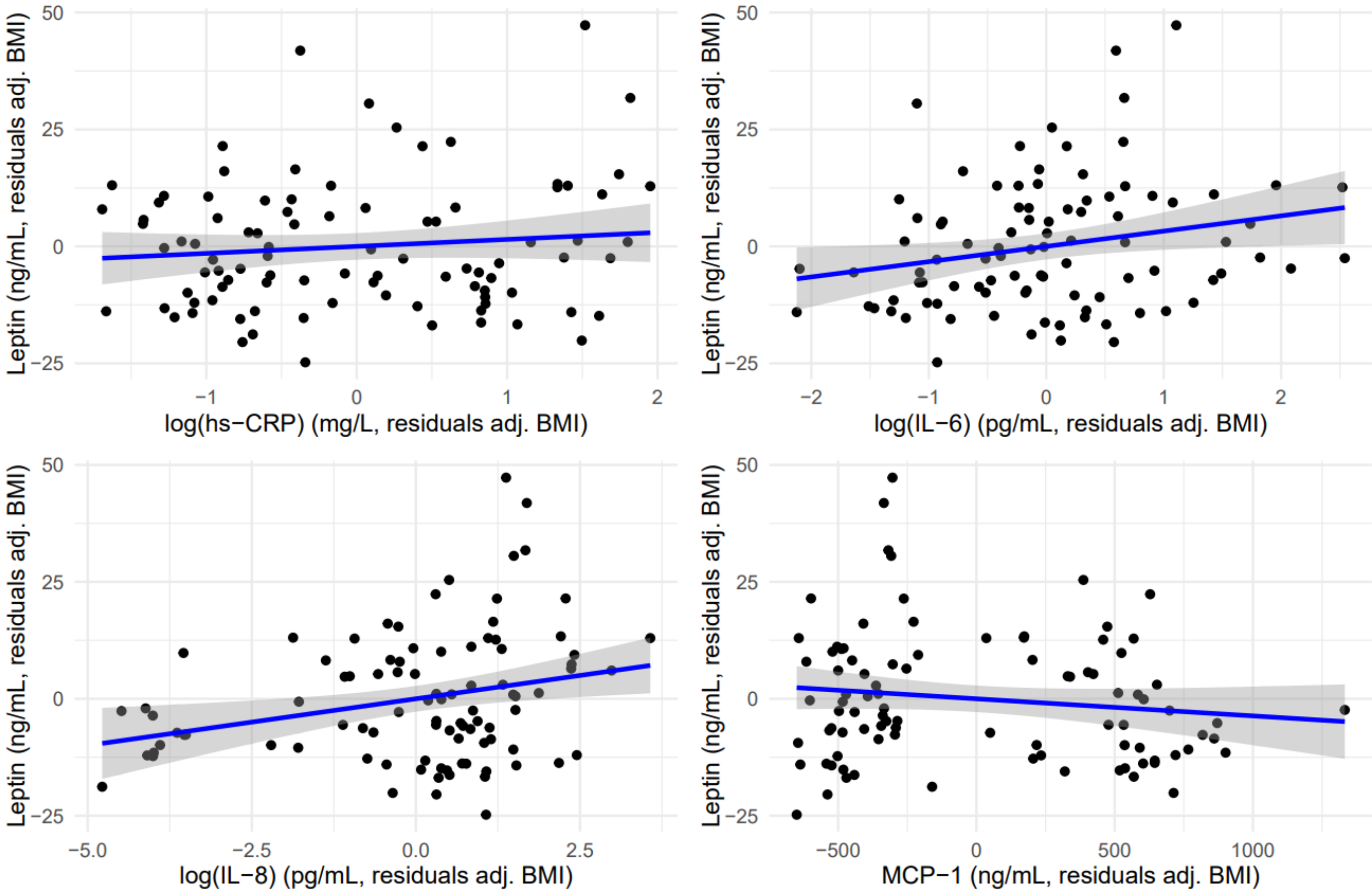

Variable	Unadjusted r (p)	Adjusted r (p)
**IL-6**	0.309 (0.00258)*	0.172 (0.101)
**IL-8**	0.317 (0.00195)*	0.245 (0.0188)*
**MCP-1**	-0.00629 (0.952)	-0.135 (0.198)
**hs-CRP**	0.347 (0.000652)*	0.193 (0.0447)*

Correlations between plasma leptin levels and inflammaging markers in people with type 2 diabetes (n=93). Linear regression graphs, adjusted for BMI, along with the Pearson's coefficients, both unadjusted and adjusted for BMI, and the relative p values showing the correlations between plasma levels of leptin and those of hs-CRP, IL-6, IL-8, and MCP-1. Hs-CRP: high-sensitivity C-reactive protein; IL: Interleukin; MCP-1: Monocyte chemoattractant protein-1. * P values < 0.05

Figure S7.

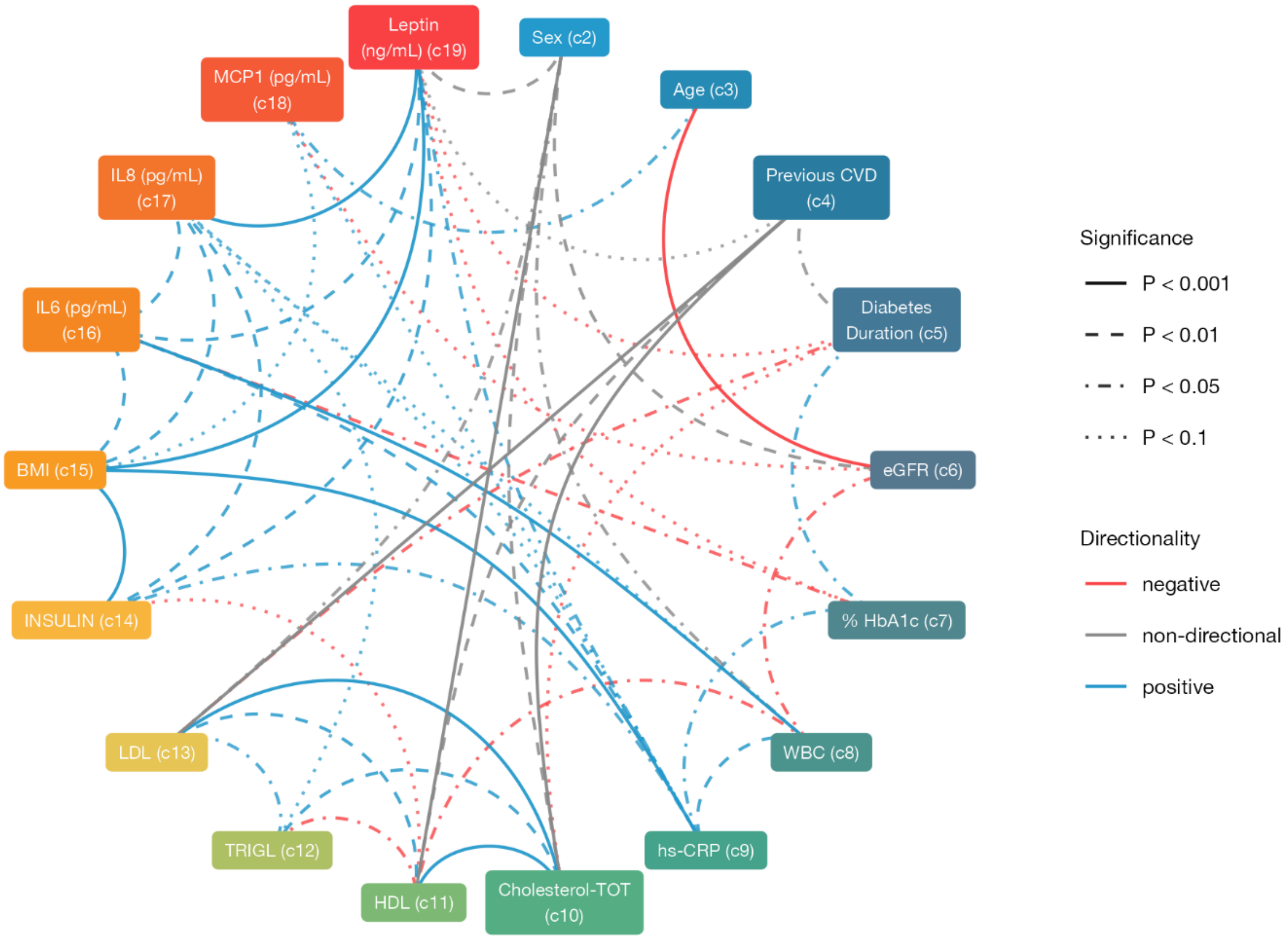

Variable network graph of correlations between multiple variables in people with type 2 diabetes. Graph summarizing all the significant relationships between leptin levels and other clinically relevant variables of the study cohort. Significance lines derive from one among Spearman correlation, Mann-Whitney test, Fisher's Exact Test, or Log-rank test tested between the variables considered, according to the type of data and their distribution. CVD: cardiovascular diseases; WBC: White blood cell counts; HbA1c: glycated hemoglobin; HDL: high density lipoprotein cholesterol; LDL: low density lipoprotein cholesterol; eGFR: estimated glomerular filtration rate; hs-CRP: high sensitivity C-reactive protein; BMI: body mass index.

Uncropped Western Blot

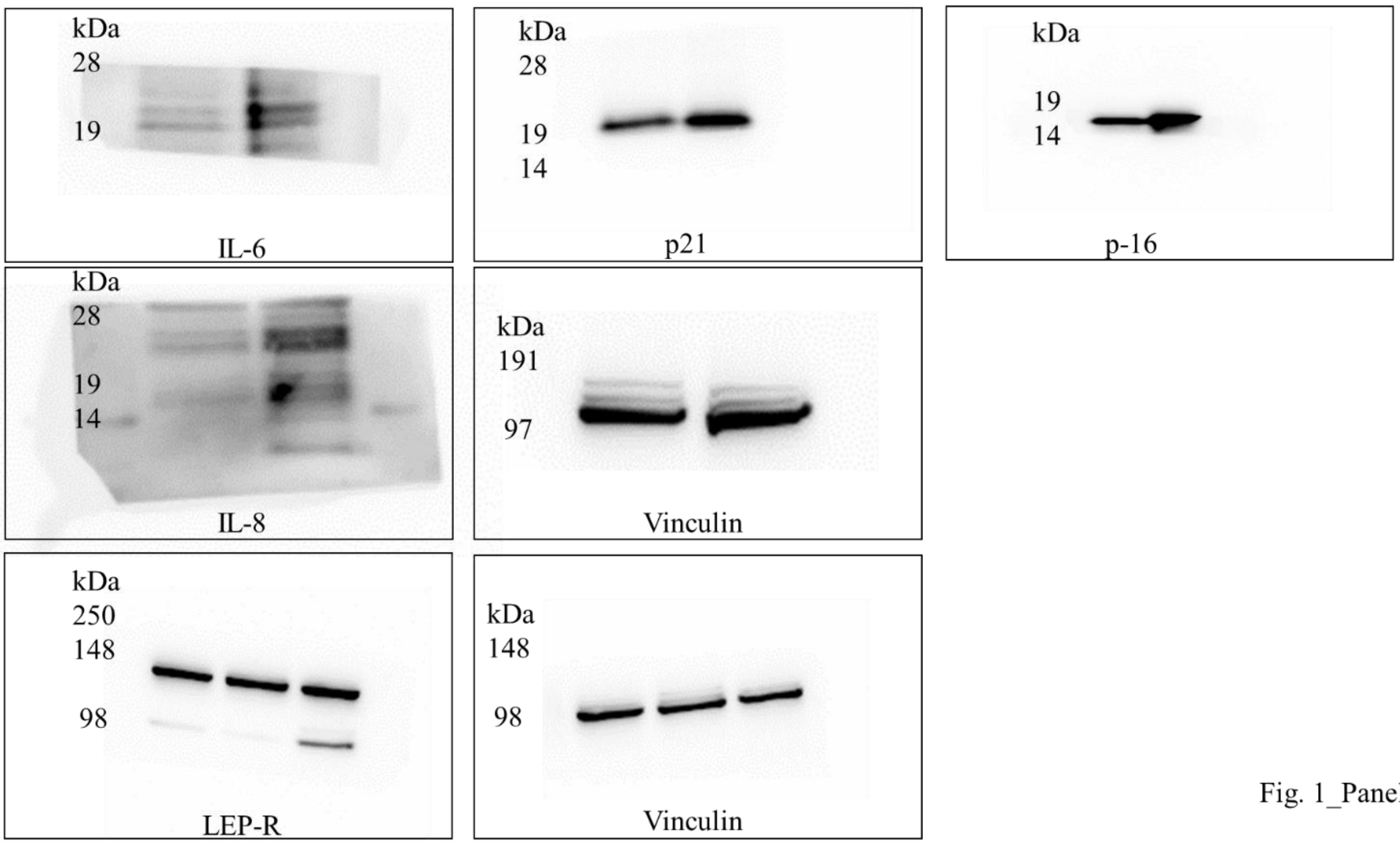

Fig. 1_Panel D

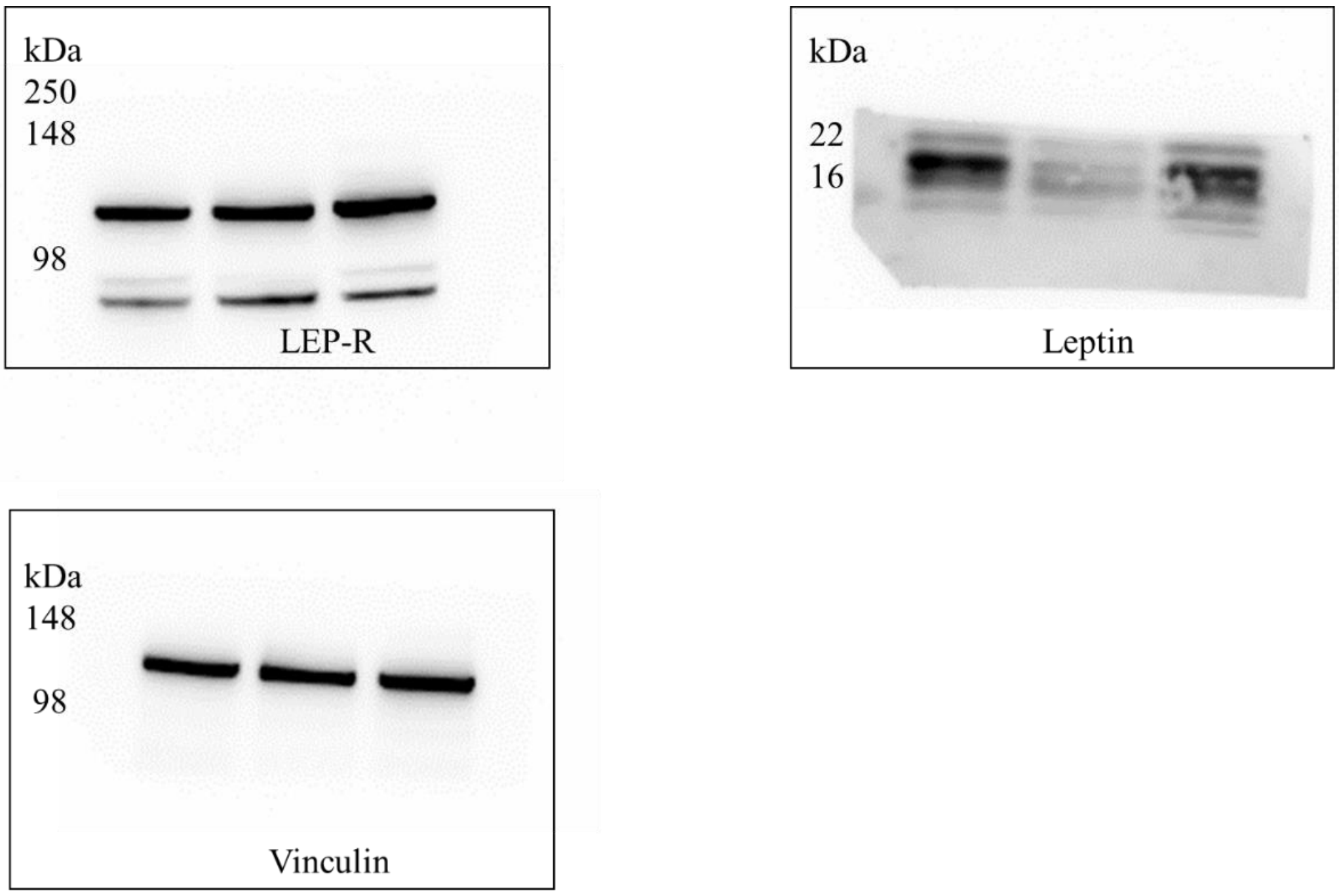

Fig. 1_Panel H

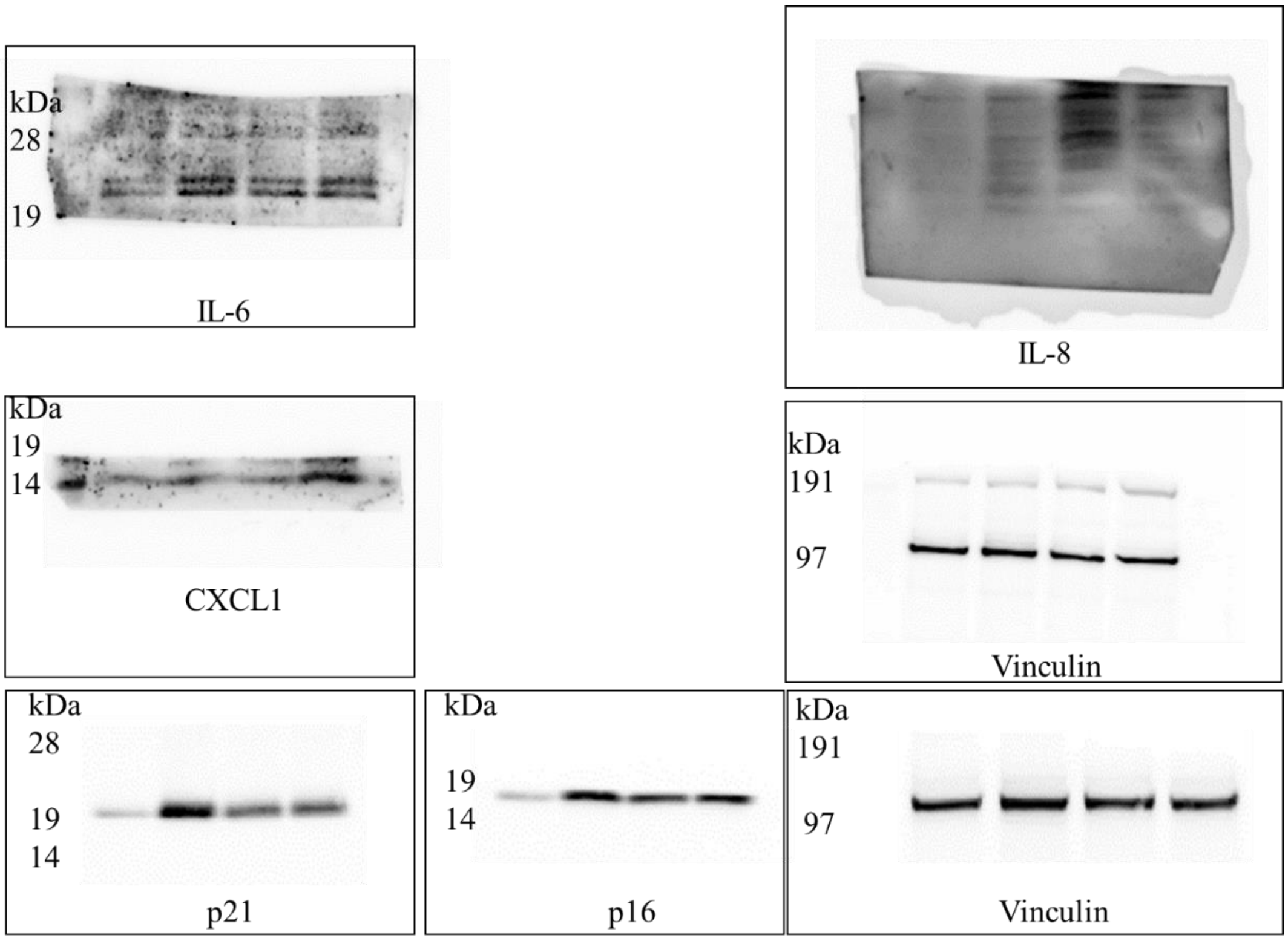

Fig. 2_Panel F

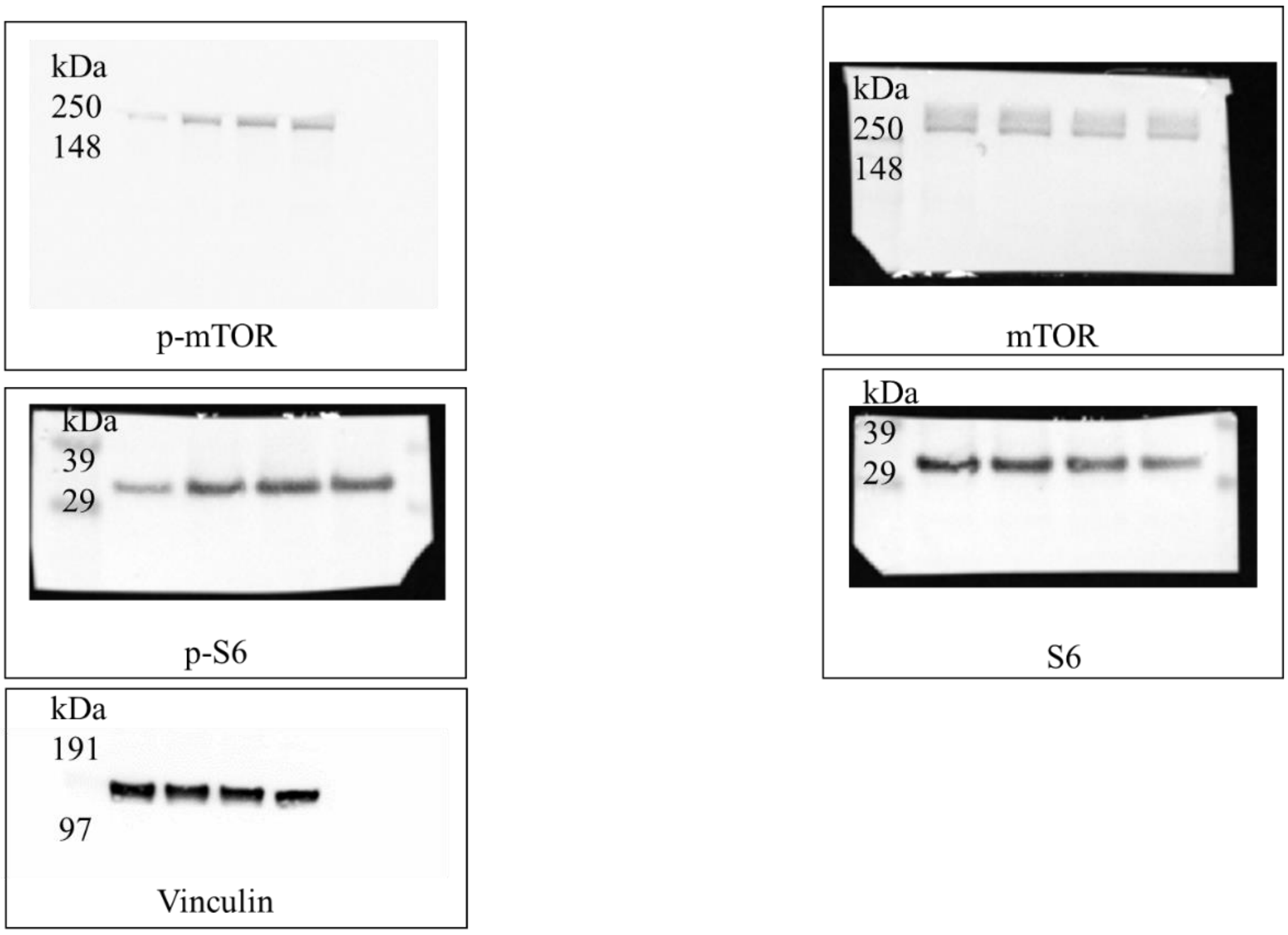

Fig. 2_Panel I

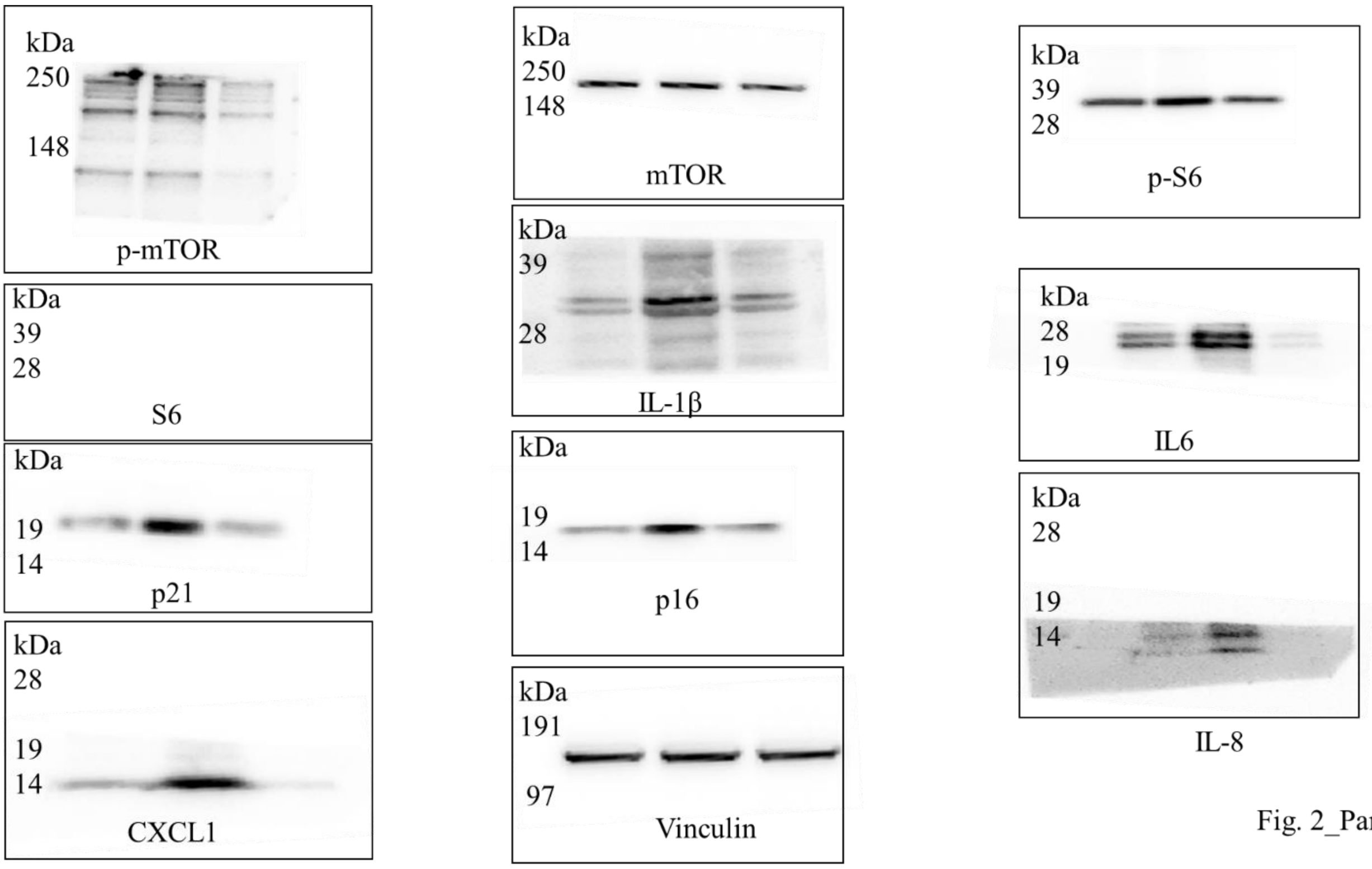

Fig. 2_Panel N

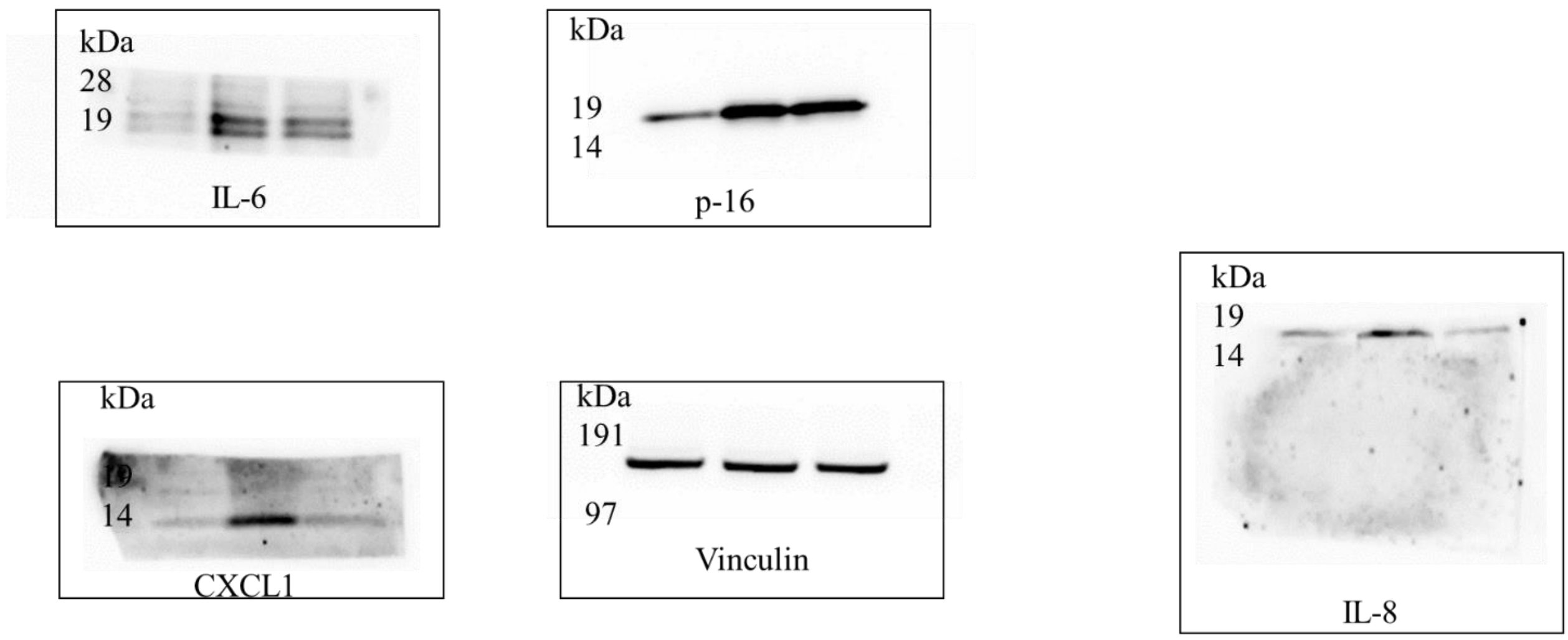

Fig. 3_Panel B

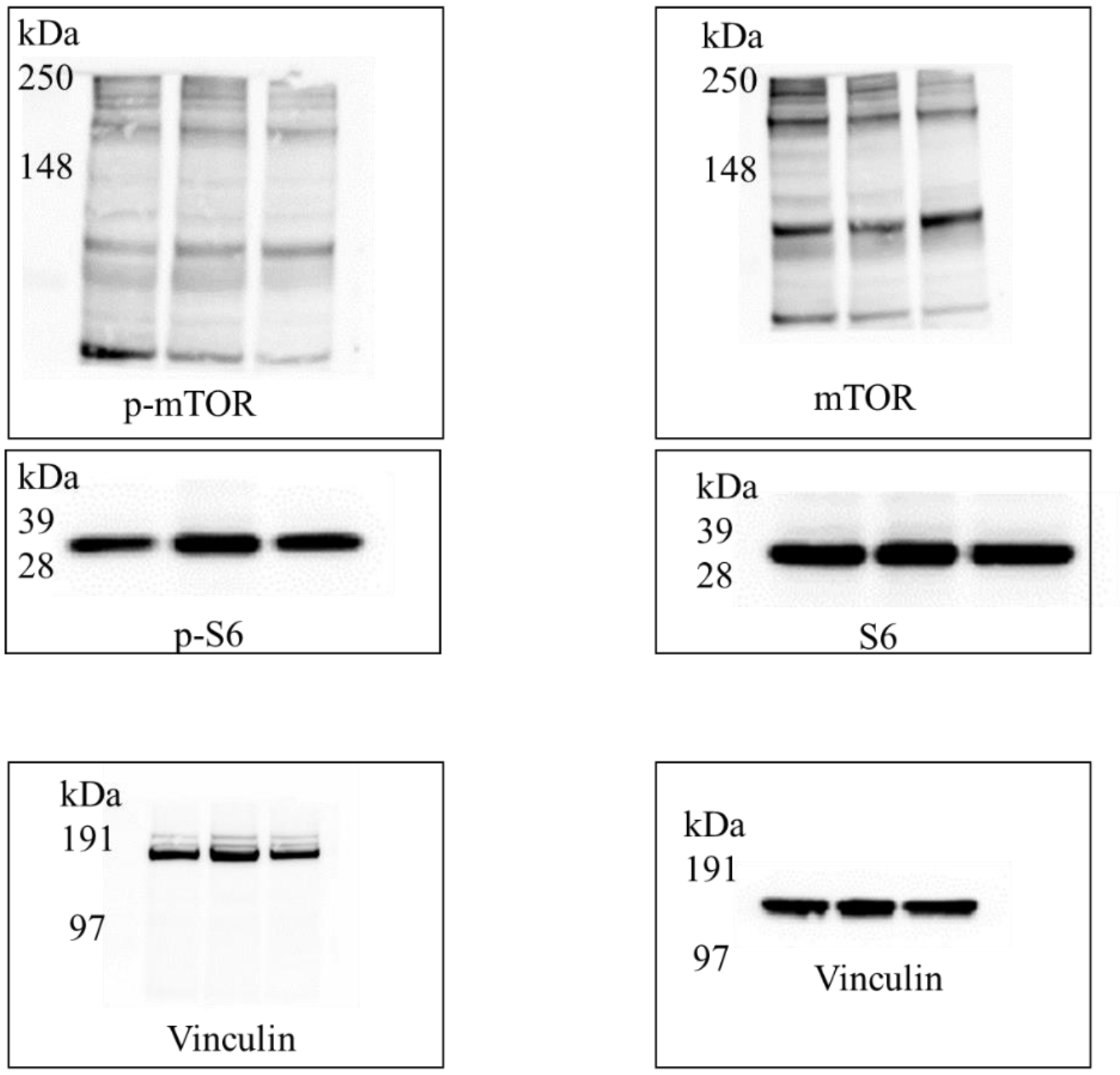

Fig. 3_Panel E

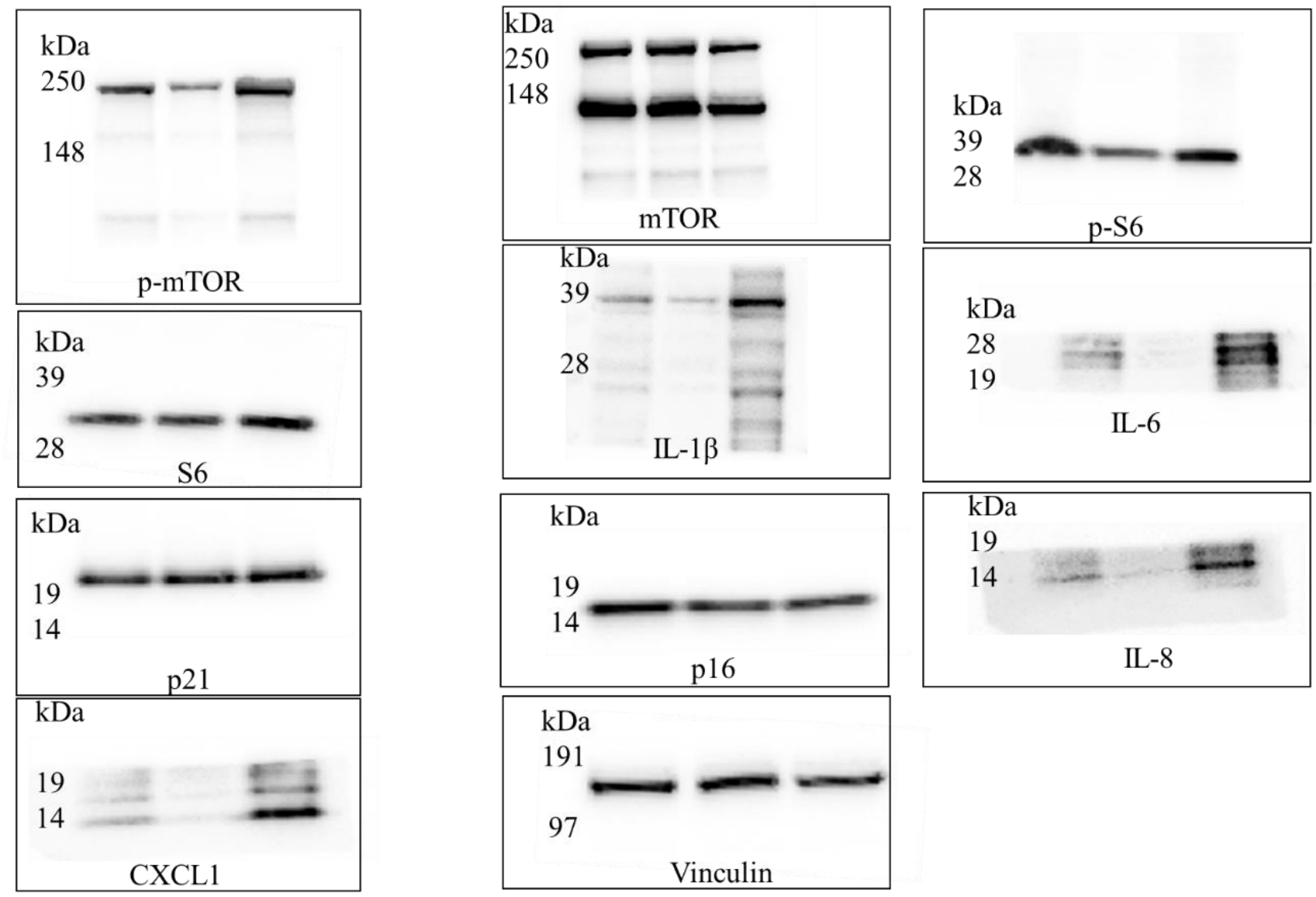

Fig. 3_Panel H

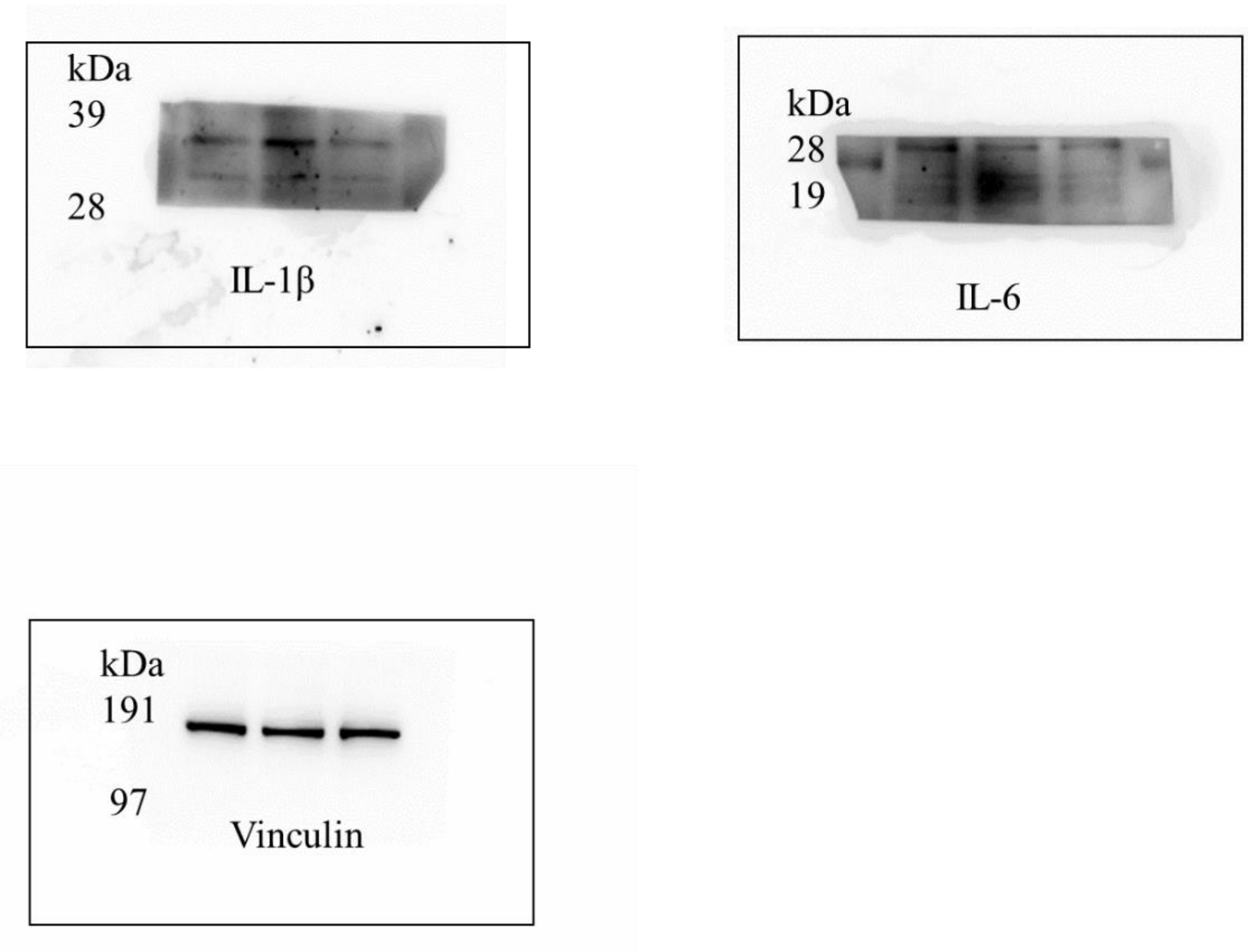

Fig. 4_Panel C

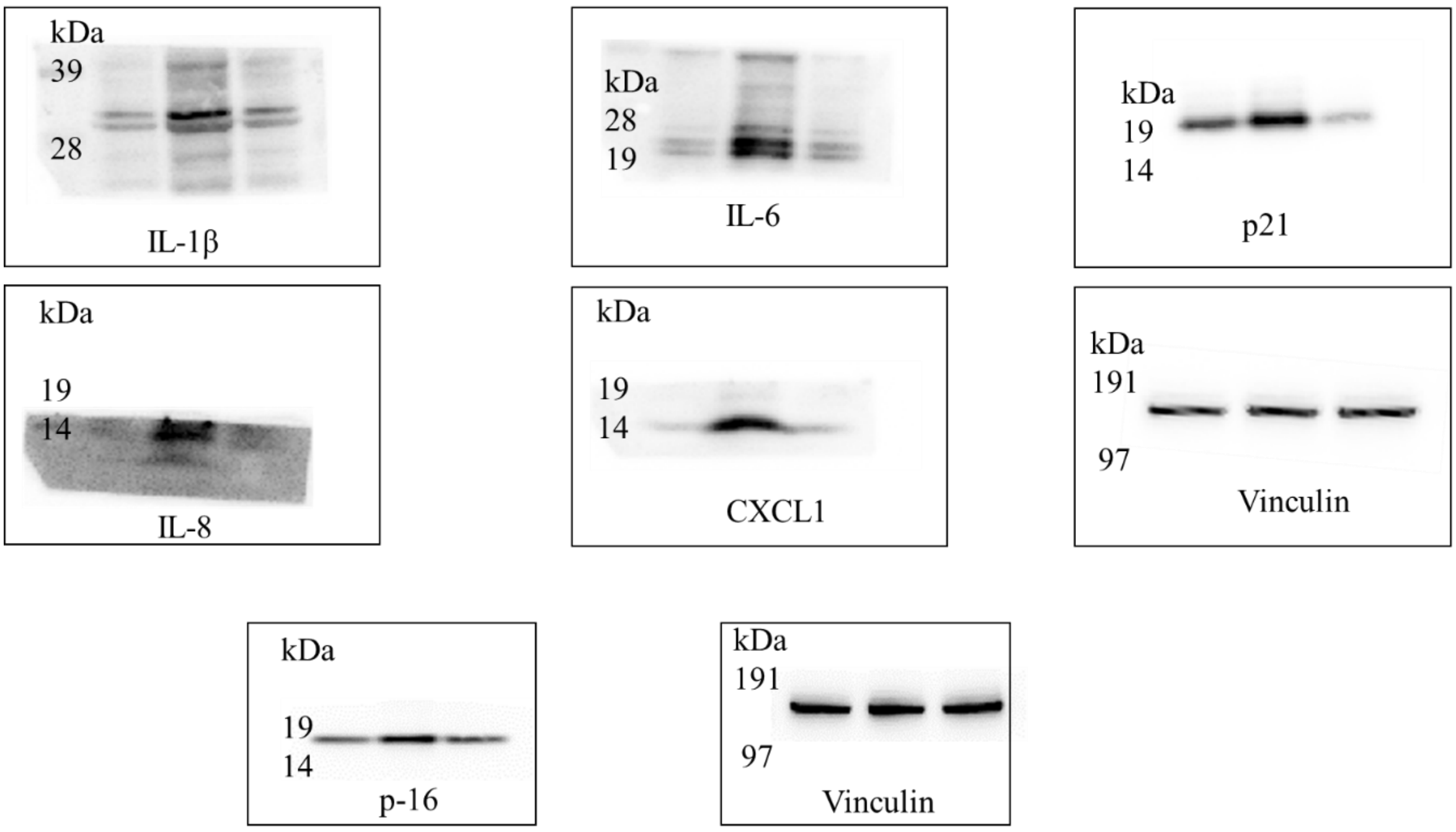

Fig. 4_Panel F

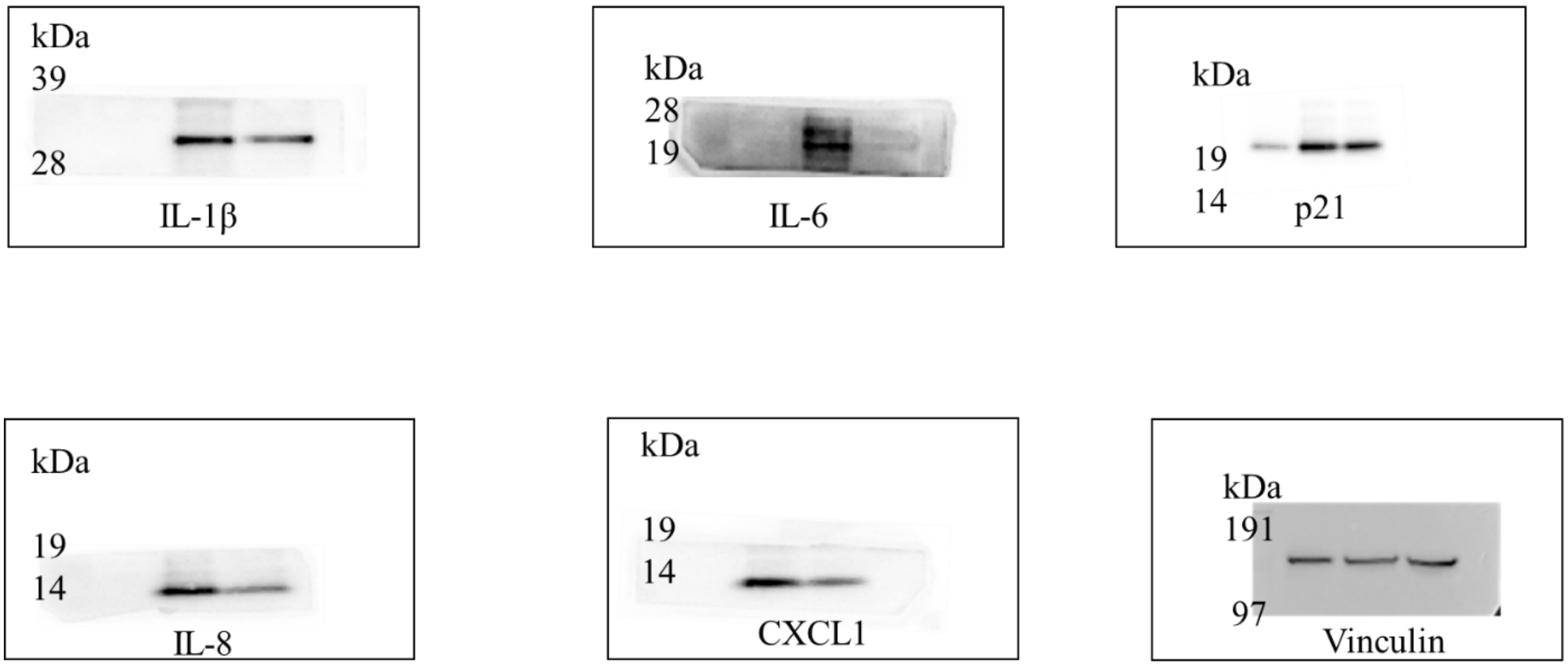

Supp Fig. 4_Panel D
